# Establishment of the neurogenic boundary of the mouse retina requires cooperation of SOX2 and WNT signaling

**DOI:** 10.1186/1749-8104-9-27

**Published:** 2014-12-09

**Authors:** Whitney E Heavner, Cynthia L Andoniadou, Larysa H Pevny

**Affiliations:** UNC Department of Genetics, University of North Carolina, Chapel Hill, NC 27599 USA; UNC Neuroscience Center, University of North Carolina, Chapel Hill, NC 27599 USA; Division of Craniofacial Development and Stem Cell Biology, King’s College London, Guy’s Hospital, Floor 27 Tower Wing, London, SE1 9RT UK; Department of Biology, Stanford University, Stanford, CA 94305 USA

**Keywords:** β-Catenin, Canonical WNT signaling, Cell cycle, Eye development, Neural progenitor cells, Proliferation, Retina, SOX2

## Abstract

**Background:**

Eye development in vertebrates relies on the critical regulation of SOX2 expression. Humans with mutations in *SOX2* often suffer from eye defects including anophthalmia (no eye) and microphthalmia (small eye). In mice, deletion of *Sox2* in optic cup progenitor cells results in loss of neural competence and cell fate conversion of the neural retina to a non-neurogenic fate, specifically the acquisition of fate associated with progenitors of the ciliary epithelium. This fate is also promoted with constitutive expression of stabilized β-Catenin in the optic cup, where the WNT pathway is up-regulated. We addressed whether SOX2 co-ordinates the neurogenic boundary of the retina through modulating the WNT/β-Catenin pathway by using a genetic approach in the mouse.

**Results:**

Upon deletion of *Sox2* in the optic cup, response to WNT signaling was expanded, correlating with loss of neural competence, cell fate conversion of the neural retina to ciliary epithelium primordium and, in addition, increased cell cycle time of optic cup progenitors. Removal of *Ctnnb1* rescued the cell fate conversion; however, the loss of neural competence and the proliferation defect resulting from lack of SOX2 were not overcome. Lastly, central *Sox2*-deficient optic cup progenitor cells exhibited WNT-independent up-regulation of D-type Cyclins.

**Conclusion:**

We propose two distinct roles for SOX2 in the developing retina. Our findings suggest that SOX2 antagonizes the WNT pathway to maintain a neurogenic fate and, in contrast, regulates cycling of optic cup progenitors in a WNT-independent manner. Given that WNT signaling acting upstream of SOX2 has been implicated in the tumorigenicity of embryonic stem cell-derived retinal progenitor cells, our results distinguish the endogenous role of WNT signaling in early optic cup patterning and support a WNT-independent role for SOX2 in maintaining retinal progenitor cell proliferation.

**Electronic supplementary material:**

The online version of this article (doi:10.1186/1749-8104-9-27) contains supplementary material, which is available to authorized users.

## Background

How neural progenitor cells maintain the ability to generate neurons over time is a major question in developmental neurobiology. The eyecup is an ideal tool to address this question, because it consists of neurogenic and non-neurogenic structures derived from a common progenitor pool originating in the eyefield of the medial anterior neural plate [1, 2]. The eyefield gives rise to the optic vesicle, which invaginates to form the optic cup (OC) around embryonic day (E) 10.0 of mouse development [3]. The OC is regionalized along the central-peripheral axis: the central OC consists of neurogenic retinal progenitor cells (RPCs) that give rise to the six neuronal and one glial cell type that make up the neural retina (NR). The peripheral OC rim, also known as the ciliary margin (CM), gives rise to the non-neurogenic epithelium of the iris and ciliary body (CB), herein referred to as ciliary epithelium (CE) (reviewed in [4]). Multipotent progenitor cells at the central boundary of the CM make a binary cell fate decision to become NR or CE [5].

Relatively little is known about the molecular mechanisms that specify neurogenic versus non-neurogenic fate in the OC. Canonical WNT signaling, which functions through its downstream transcriptional effector β-Catenin, has been identified as a major regulator of CE fate specification in many species [6–12]. In the chick eye, WNT/β-Catenin signaling was found to inhibit NR fate and promote CE fate [7]. In the mouse, a genetic reporter under the control of β-Catenin/TCF/LEF response elements showed WNT activity to be concentrated to the CM [6], and specific ablation of *Ctnnb1* in OC progenitor cells (OCPCs) reduced the size of the CE progenitor cell pool [8, 13]. Conversely, stabilized expression of *Ctnnb1* in mouse OCPCs induced ectopic expression of CE-specific genes [8]. However, these ectopic CE-like cells did not express *Pax6* or *Chx10*, two well-known transcriptional regulators of CE fate, suggesting that there was only a partial transformation of NR-to-CE upon activation of *Ctnnb1*.

Unsurprisingly, in light of the above, removing the inhibition of WNT signaling also induced CE fate; *Foxg1*-null embryos showed expanded WNT activity into the central OC and ectopic formation of CE positive for both *Pax6* and *Chx10*, suggesting that near physiological levels of β-Catenin may be required to generate all the hallmarks of CE [14]. Thus, precise regulation of the level of WNT activity is crucial for establishing the proper boundary between CE and NR, where factors mediating this function are not known.

SRY (sex determining region Y)-box (SOX) proteins are known regulators of WNT signaling in several developmental systems and disease states. SOX2, a member of the SOXB1 family of transcription factors, is a major regulator of neural competence in vertebrates [15–17]. Heterozygous mutations in human *SOX2* are associated with anophthalmia (absent eye) and account for 10 to 20% of cases of severe bilateral ocular malformation, including microphthalmia (small eye) [18–20] indicating a defect in OCPC proliferation or survival. In the mouse OC, SOX2 expression is restricted to the presumptive NR, and ablation of *Sox2* in OCPCs resulted in loss of neural competence and cell fate conversion of the NR to CE primordium, accompanied by an increase in WNT signaling [5]. The genetic relationship between SOX2 and WNT signaling in this context was not investigated.

In addition to eye defects, human patients with *SOX2* mutations often have pituitary abnormalities, and WNT signaling is known to be involved in hypothalamic and pituitary development. Human SOX2 protein can inhibit β-Catenin-driven reporter expression *in vitro*, but several SOX2 proteins with the identified human mutations cannot. Therefore, it has been suggested that an inability to repress WNT/β-Catenin signaling may contribute to the pathogenesis of *SOX2* loss-of-function (LOF) mutations in human patients [21, 22]. In support of this hypothesis, a SOX2 binding site was identified in the *Lef1* promoter and was found to function as a repressor of β-Catenin-dependent *Lef1* expression in primary airway epithelial cells [23]. Additionally, in osteoblasts, SOX2 was shown to physically associate with β-Catenin to down-regulate the expression of many WNT target genes, but the HMG domain was not required, suggesting that SOX2 may antagonize WNT signaling via β-Catenin sequestration [24].

The complementary eye phenotypes associated with *Sox2* and *Ctnnb1* LOF suggest antagonism between these two pathways in mammalian OC development. In lower vertebrates and in RPCs differentiated from induced pluripotent stem cells, these two pathways have been found to work somewhat synergistically to promote retinal neural progenitor proliferation [25, 26]. These findings may reflect species-specific differences in the role of WNT signaling in OC development. Alternatively, WNT signaling may play different roles over developmental time: constitutive activation of WNT signaling later in development, in a subset of committed neural precursors, may have different effects than that of widespread WNT activation at earlier time points, in uncommitted OCPCs. Given the evidence that SOX2 and WNT signaling play complex and crucial roles in the eye development of many species, we chose to dissect the relationship between these two factors using a genetic approach in the mouse.

In this study, we investigated the hypothesis that SOX2 antagonizes canonical WNT signaling to maintain neurogenic fate in the mouse OC. We present whole-genome expression arrays comparing wild-type and *Sox2*-mutant OCs demonstrating the deregulation of the WNT pathway and serving as a resource for identifying genes involved in CE fate and function, with direct relevance to understanding the pathogenesis of diseases associated with the anterior segment. We show that removal of *Ctnnb1* from the *Sox2*-mutant OC partially rescued the *Sox2*-mutant phenotype. Conversely, ectopic activation of the WNT pathway in *Sox2*-expressing cells resulted in acquisition of CE fate following loss of *Sox2* expression. Our data provide evidence that SOX2 antagonizes CE fate via modulation of WNT signaling and highlight a β-Catenin-independent role for SOX2 to promote proliferation and prevent aberrant expression of cell cycle regulators in OCPCs.

## Results

### Canonical WNT signaling is ectopically activated in *Sox2*-mutant optic cups

**Figure 1:**
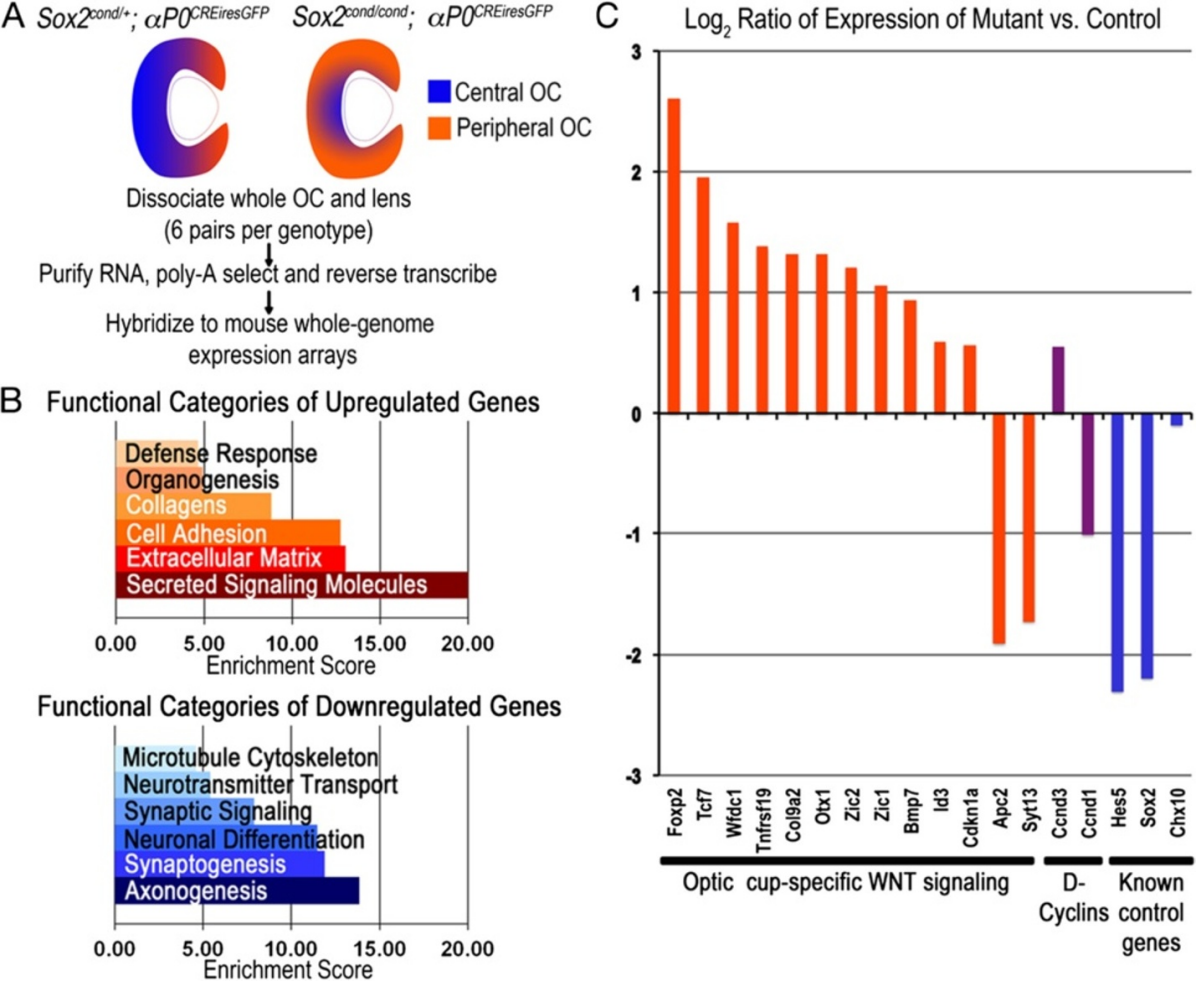
**Genome-** **wide expression analysis of E16.5** ***Sox2***-**mutant optic cups (OCs) confirms loss of neural retina (NR) fate,** **activation of ciliary epithelium (CE) primordium genes**, **and suggests activated WNT signaling.** Global changes in gene expression in *Sox2*-ablated OCs undergoing NR-to-CE primordium cell fate conversion were analyzed by microarray. **(A)** Diagram of expansion of the peripheral OC/prospective CE (orange) at the expense of the central OC/prospective NR (blue) in mutants compared with controls. **(B)** Ontologies of increased transcripts (orange) are consistent with CE function, while those of decreased transcripts (blue) are consistent with NR function. **(C)** Subsets of aberrantly expressed genes suggest activated WNT signaling and perturbed cell cycle. Decreased *Hes5* and *Sox2*, and unchanged *Chx10*, confirm the validity of the screen.

Ablation of *Sox2* in the mouse OC from E10.5 leads to eventual loss of NR fate and expansion of the non-neurogenic CE [5]. To determine the molecular mechanisms underlying this phenotype, we performed a whole genome expression screen of *Sox2*^*cond*/+^;*αP0*^*CREiresGFP*^ ('control') and *Sox2*^*cond*/*cond*^;*αP0*^*CREiresGFP*^ ('mutant') eyes at E16.5, when the loss of neural fate is taking place (Figure 1A). We ran one microarray for each of six pairs of eyes per genotype, for a total of twelve microarrays. The complete results from this screen have been deposited in NCBI’s Gene Expression Omnibus [27] and can be accessed through the GEO series accession number GSE46796 (http://www.ncbi.nlm.nih.gov/geo/query/acc.cgi?acc=GSE46796). We identified 880 significantly up-regulated genes and 951 significantly down-regulated genes in mutant OCs compared with controls (see Methods). To confirm the efficacy of this screen, we first verified that transcripts found to change by *in situ* hybridization (ISH) [5] behaved as expected by microarray (Figure 1C). As anticipated, in mutants compared with controls, *Sox2* was decreased over 4-fold, *Hes5*, an NR marker, which was found to be absent in *Sox2*-ablated cells, was decreased almost 5-fold, and *Chx10*, a definitive marker of OC progenitors at this stage, and which was shown to be maintained, was not significantly changed.

We then used DAVID analysis software [28, 29] to search for enriched functional terms in each group of genes and determine if they were consistent with a change in cell fate. The full list of genes for each enriched functional term for each group is provided in Additional file 1. Up-regulated genes were enriched for terms associated with secreted signaling, extracellular matrix, cell adhesion, organogenesis and the defense response, all of which are consistent with the function of the CE to maintain the intraocular pressure through secretion [30, 31] and to produce proteins of the inner limiting membrane and the vitreous body [32, 33] (Figure 1B). By contrast, down-regulated genes were enriched for terms associated with axonogenesis, synaptic development, neuronal differentiation and microtubule cytoskeleton, consistent with the loss of neurogenic fate observed in mutants. Collectively, these data confirm that our screen provides a reliable indication of functional pathways disrupted by *Sox2* ablation.

We next queried which signaling pathways were disrupted in *Sox2*-mutant eyes. Two of the most significantly affected pathways were canonical WNT signaling and transforming growth factor beta (TGFβ) signaling, both known for their involvement in CE fate specification [7, 8, 34, 35]. We focused further on WNT signaling, given that SOX2 was shown to antagonize the pathway in the *Xenopus* OC [25], and we have observed ectopic expression of WNT pathway genes, including *Lef1*, *Sfrp2* and *Axin2*, in the *Sox2*-mutant OC at E14.5 [5]. We scanned our set of significantly changed transcripts for known OC-specific WNT target genes and identified 13 for which expression was changed at least 1.47-fold (Figure 1C, orange bars; Additional file 2) [9, 11, 12, 36, 37]. We also confirmed that *Axin2*, a well-characterized read-out of WNT signaling, was increased (1.29-fold, *P* = 0.0155). Collectively, these data suggest that response to the WNT pathway was activated upon *Sox2* ablation. We thus hypothesized that SOX2 normally antagonizes canonical WNT signaling to maintain neurogenic fate in the mouse OC.

### Genetic removal of *Ctnnb1*does not rescue the neurogenesis defect of *Sox2*-mutant retinas

If increased WNT signaling causes loss of neural competence upon ablation of *Sox2*, then reduction of WNT activity should rescue neurogenesis in *Sox2*-mutant eyes. SOX2 protein has been shown to interact directly with β-Catenin/*Ctnnb1*, the main transcriptional mediator of canonical WNT signaling, to inhibit the activation of target genes in many biological systems [24, 38–40]. So to address this hypothesis, we performed epistasis analysis using conditional alleles of both *Sox2* and *Ctnnb1* an OC-specific Cre. *Ctnnb1*^*lox*(*ex2*–*6*)/+^ mice, in which exons 2 to 6 of the *Ctnnb1* locus are flanked by *loxP* sites [41], were crossed with transgenic mice carrying *Chx10*^*CreGFP*^, a BAC transgene expressing Cre recombinase in OCPCs as early as E10.5 [42]. For this experiment, we chose to use *Chx10*^*CreGFP*^ in place of *αP0*^*CreiresGFP*^ in order to avoid differential regulation of the αP0Cre transgene in the absence of SOX2 and β-Catenin, as previously observed [5, 8]. Distinctions between our uses of *αP0*^*CreiresGFP*^ and *Chx10*^*CreGFP*^ are summarized in Table 1.

**Table 1:** **Distinctions between the two optic cup (OC)**-**specific Cre lines used in this study**

CRE line	Onset	Expression pattern	Applications in this study
*αP0* ^*CREiresGFP*^	E10.5	Prospective CE and NR (Peripheral^HI^ - Central^LO^)	Whole genome expression screen; Proliferation analysis
*Chx10* ^*CRE*-*GFP*^	E10.5	Prospective CE and NR (Mosaic)	Genetic epistasis (Cre expression is not affected by SOX2 or β-Catenin)

*Abbreviations*: *CE* ciliary epithelium, *NE* neural retina.

*Ctnnb1*^*lox*(*ex2*–*6*)/+^;*Chx10*^*CreGFP*^ mice were crossed with *Sox2*^*cond*/+^ mice to produce *Sox2*/*Ctnnb1* double-mutant OCPCs. We compared the eyes of *Sox2* single-mutants (*Sox2*^*cond*/*cond*^;*Chx10*^*CreGFP*^) and *Ctnnb1* single-mutants (*Ctnnb1*^*lox*(*ex2*–*6*)/*lox*(*ex2*–*6*)^;*Chx10*^*CreGFP*^) with *Sox2*/*Ctnnb1* double-mutants (*Sox2*^*cond*/*cond*^;*Ctnnb1*^*lox*(*ex2*–*6*)/*lox*(*ex2*–*6*)^;*Chx10*^*CreGFP*^) and stage-matched controls (*Sox2*^*cond*/+^;*Ctnnb1*^*lox*(*ex2*–*6*)/+^;*Chx10*^*CreGFP*^).

We first verified that *Sox2* and *Ctnnb1* were efficiently ablated with *Chx10*^*CreGFP*^ and that co-ablation did not increase cell death (Additional file 3). In double-mutant OCs, SOX2 and β-Catenin proteins were absent from GFP-positive cells, demonstrating that *Chx10*^*CreGFP*^ was able to recombine all floxed alleles in the same nuclei (Additional file 3D, E versus A, B). Moreover, deletion of both genes did not increase cell death as assayed by activated-Caspase 3 staining (Additional file 3C, F).

**Figure 2:**
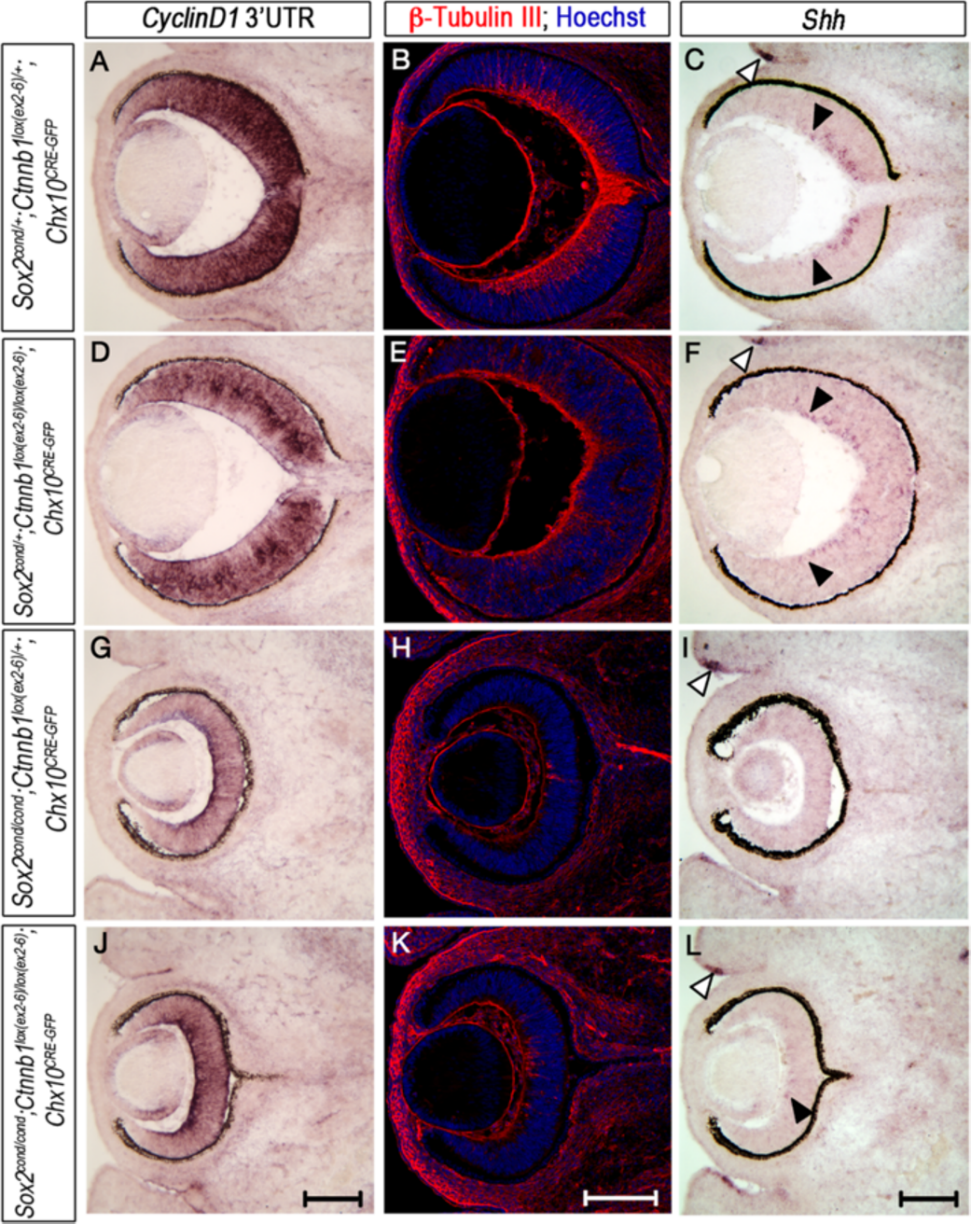
**Deletion of** ***Ctnnb1*** **does not restore neurogenesis to** ***Sox2-*** **mutant cells.** Transverse sections of E13.5 control and mutant eyes were analyzed for restoration of neurogenic fate to *Sox2*-ablated regions. **(A, D, G, J)** *Ccnd1* mRNA localizes to the central optic cup (OC) of wild-type controls **(A)** and *Ctnnb1* ^*lox*(*ex2*–*6*)/*lox*(*ex2*–*6*)^ single-mutants **(D)** and is maintained in the central OC of *Sox2* single-mutants **(G)** and *Sox2*/*Ctnnb1* double-mutants **(J)**. **(B, E, H, K)** β-Tubulin III, marking neurons, is expressed in the retinal ganglion cell (RGC) layer of controls **(B)** and *Ctnnb1* single-mutants **(E)**, but its expression is markedly reduced in *Sox2* single-mutant **(H)** and double-mutant **(K)** cells. **(C, F, I, L)** Sonic Hedgehog (*Shh*) mRNA expression correlates with the pattern of β-Tubulin III staining, and thus is significantly reduced in mutants **(I, L)** compared with controls **(F, C)**. Black arrowheads in **(C)** and **(F)** indicate the limits of *Shh* expression. White arrowheads in **(C,** **F, I and** **L)** point to an unaffected region of *Shh* expression in the presumptive eyelid. Scale bars: 200 μm.

To test the hypothesis that removal of *Ctnnb1* from *Sox2*-mutant cells would rescue the loss of neural competence, we examined expression of prospective NR markers in single-mutant and double-mutant OCs at E13.5 (Figure 2; Table 2). We first examined expression of β-Tubulin III, a neuronal marker, to determine whether neurogenesis was restored in double- mutant OCs. β-Tubulin III was expressed in the central OC of controls, marking retinal ganglion cells (RGCs), but was broadly lost in *Sox2* single-mutants and in *Sox2*/*Ctnnb1* double-mutants (Figure 2B and E versus H and K). RGCs express Sonic Hedgehog (*Shh*), so to confirm the observed loss of neurogenesis, we examined *Shh* expression in control and mutant eyes. Like β-Tubulin III, *Shh* was expressed in the RGC layer of controls (Figure 2C, F, area between closed arrowheads), but expression was essentially lost in *Sox2* single-mutant and double-mutant eyes (Figure 2I, L, closed arrowhead indicating residual *Shh* in a double-mutant eye correlating with residual β-Tubulin III expression). *Shh* expression in the presumptive eyelids remained unchanged in all genotypes, confirming the efficacy of the staining (Figure 2C, F, I, L, white arrowheads).

**Table 2:** **Differences in the expression patterns of central optic cup progenitor cells (OCPCs) of wild**-**type**, **single**-**mutant and double**-**mutant eyes**

	NR markers		Ciliary epithelium markers				Optic cup progenitor cell TFs			D-Cyclins (protein)	
Genotype	***Hes5***	β-Tubulin/ ***Shh***	***Msx1***	***Otx1***	***BMP4***	***Zic1***	***Chx10***	***Rax***	***Pax6***	CyclinD3	CyclinD1
Wild-type	expressed	expressed	*absent*	*absent*	*absent*	*absent*	expressed	expressed	expressed	*absent*	expressed
Sox2^-/-^; Ctnnb1^-/+^	*absent*	*absent*	expressed	expressed	expressed	expressed	expressed	expressed	**increased**	expressed	**increased**
Sox2^-/+^; Ctnnb1^-/-^	expressed	expressed	*absent*	*absent*	*absent*	*absent*	expressed	expressed	expressed	*absent*	expressed
Sox2^-/-^; Ctnnb1^-/-^	*absent*	*absent*	*absent*	*absent*	*absent*	*absent*	expressed	expressed	**increased**	expressed	**increased**

*Abbreviation*: *TFs* transcription factors.

CyclinD1/*Ccnd1* is normally restricted to the central OC and is often used to mark NR progenitor cells [8, 14]. In wild-type controls and *Ctnnb1* single-mutant eyes, *Ccnd1* mRNA was expressed in central neurogenic progenitor cells. *Ccnd1* expression persisted in *Sox2* single-mutant OCs despite the loss of NR fate (Figure 2A and D versus G). Overall, *Ccnd1* staining appeared decreased in *Sox2*-ablated cells consistent with the gene expression results of the microarray analysis, which showed a 2-fold decrease (*P* = 0.001). Moreover, removal of *Ctnnb1* from *Sox2*-mutant OCPCs did not further diminish *Ccnd1* expression (Figure 2J). In summary, genetic reduction of WNT activation did not restore neural competence to *Sox2*-ablated eyes at E13.5, and expression of *Ccnd1* was sustained despite loss of either pathway.

### Genetic ablation of β-Catenin prevents cell fate conversion of *Sox2*-mutant retinas

**Figure 3:**
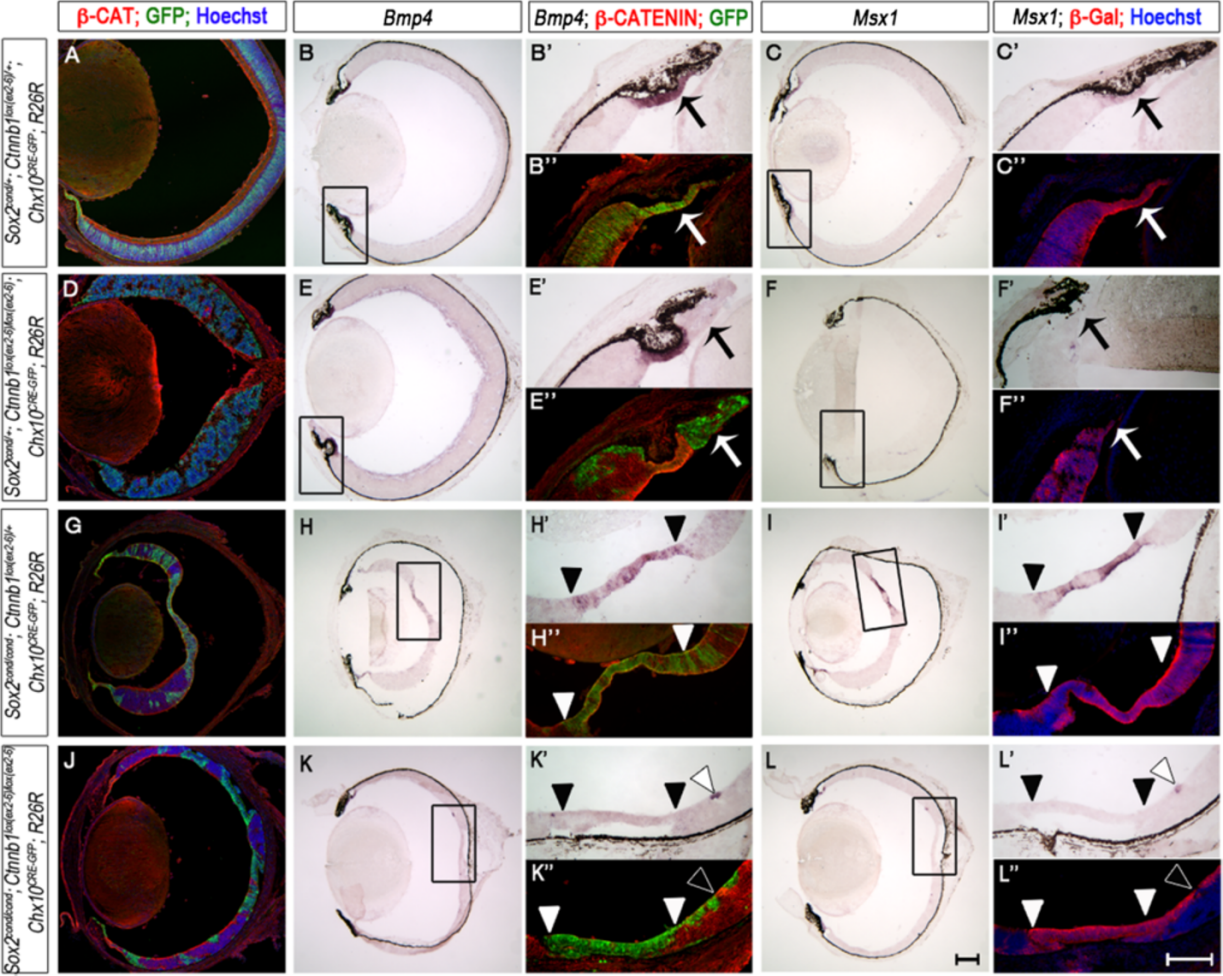
**Deletion of** ***Ctnnb1*** **prevents the ectopic expression of ciliary epithelium (CE) genes in** ***Sox2-*** **mutant cells.** Transverse sections of P0 eyes were analyzed for CE fate. **(A, D, G, J)** Low magnification view of β-Catenin/GFP-stained eyes indicates hypoplasia of *Sox2* single-mutants **(G)** and *Sox2*/*Ctnnb1* double-mutants **(J)** compared with *Ctnnb1* ^*lox*(*ex2*–*6*)/*lox*(*ex2*–*6*)^ single-mutants **(D)** and wild-type littermates **(A)**. **(B** ***-*** **C** ***”*** **)** *In situ* hybridization of *Bmp4* **(C)** and *Msx1* **(D)** shows specific expression of these genes in GFP-positive **(B”)** and β-galactosidase (β-gal)-positive **(C”)** CE of wild-type controls. **(E-F”)** *Bmp4* **(E)** and *Msx1* **(F)** are not expressed in the GFP-positive (**E**”, arrow**)** and β-gal-positive (**F**”, arrow) CE of *Ctnnb1* single-mutants. **(H-I”)** Conversely, *Bmp4* **(H)** and *Msx1* **(I)** are ectopically expressed in the GFP-positive (**H**”, closed arrowheads) and β-gal-positive (**I”**, closed arrowheads) central optic cup (OC) of *Sox2* single-mutants. **(K-L”)** Ectopic expression of *Bmp4* **(K)** and *Msx1* **(L)** is rescued in the GFP-positive (**K**”, closed arrowheads) and β-gal-positive (**L**”, closed arrowheads) central OC of *Sox2*/*Ctnnb1* double-mutants. Open arrowheads indicate faint expression of CE genes at the periphery of the NR of double-mutants. Boxes in **(** **B-C)**, **(E-F)**, **(H-I)** and **(K-** **L)** are magnified in ’ and ”. Scale bars: 100 μm.

We next examined single- and double-mutant neonatal eyes to determine whether removal of *Ctnnb1* could rescue the NR-to-CE cell fate conversion previously observed in *Sox2*-mutant eyes [5]. At P0, *Sox2* single-mutant and *Sox2*/*Ctnnb1* double-mutant eyes were small, and the optic neuroepithelium of both was hypoplastic compared with controls (Figure 3A, B and E, F versus I, J and M, N). To clearly identify mutant cells in each background, we applied two methods: first we used an antibody against β-Catenin to distinguish positive and negative cells (Figure 3B”, E”, H”, K”). Secondly, we backcrossed all genotypes to reporter mice expressing *β*-*galactosidase* (*β*-*gal*) from the *ROSA26* locus [43] to mark recombined cells (Figure 3C”, F”, I”, L”).

*Bmp4* and *Msx1* were expressed in the presumptive CE of wild-type controls as expected (Figure 3B, C) [8, 44]. By contrast, *Bmp4* and *Msx1* were absent from presumptive CE cells of *Ctnnb1* single-mutants, supporting the hypothesis that canonical WNT signaling directly regulates the expression of CE primordium genes (Figure 3E, F, E’ and F’ arrows). As seen in our previous study, *Bmp4* and *Msx1* were ectopically expressed in *Sox2*-ablated regions (Figure 3H, I, H’ and I’, area between closed arrowheads) [5]. This ectopic up-regulation of *Bmp4* and *Msx1* occurred in approximately 30 to 60% of recombined cells based on GFP or β-gal expression. Strikingly, genetic deletion of *Ctnnb1* from these cells prevented the ectopic expression of *Bmp4* and *Msx1* (Figure 3K, L, K’, L’ area between closed arrowheads) in all retinas analyzed (n = 3 per genotype, per stage). We noted marginal CE gene expression at the periphery of β-Catenin-positive/Cre-negative regions in double-mutants (Figure 3 K’, L’ open arrowheads), possibly indicating a signaling center for CE specification at the edges of NR, as has been reported in the chick OC [45].

We next examined expression of additional NR and CE markers in control, single-mutant and double-mutant OCs at P0, and these results are summarized in Table 2. Similar to *Bmp4* and *Msx1*, ectopic expression of the CE genes *Otx1* and *Zic1* was rescued in double-mutants. The pan-OCPC transcription factors *Chx10* [42] and *Rax* [46] were unchanged in all genotypes. Notably, *Pax6*, which was increased in *Sox2* single-mutants [5], remained so in double-mutants, suggesting that SOX2 antagonizes *Pax6* independently of WNT signaling. Together, these data suggest that SOX2 antagonizes CE identity, in part through mediation of WNT/β-Catenin activity.

### Ectopic WNT pathway activation in SOX2-expressing cells results in loss of NR fate

**Figure 4:**
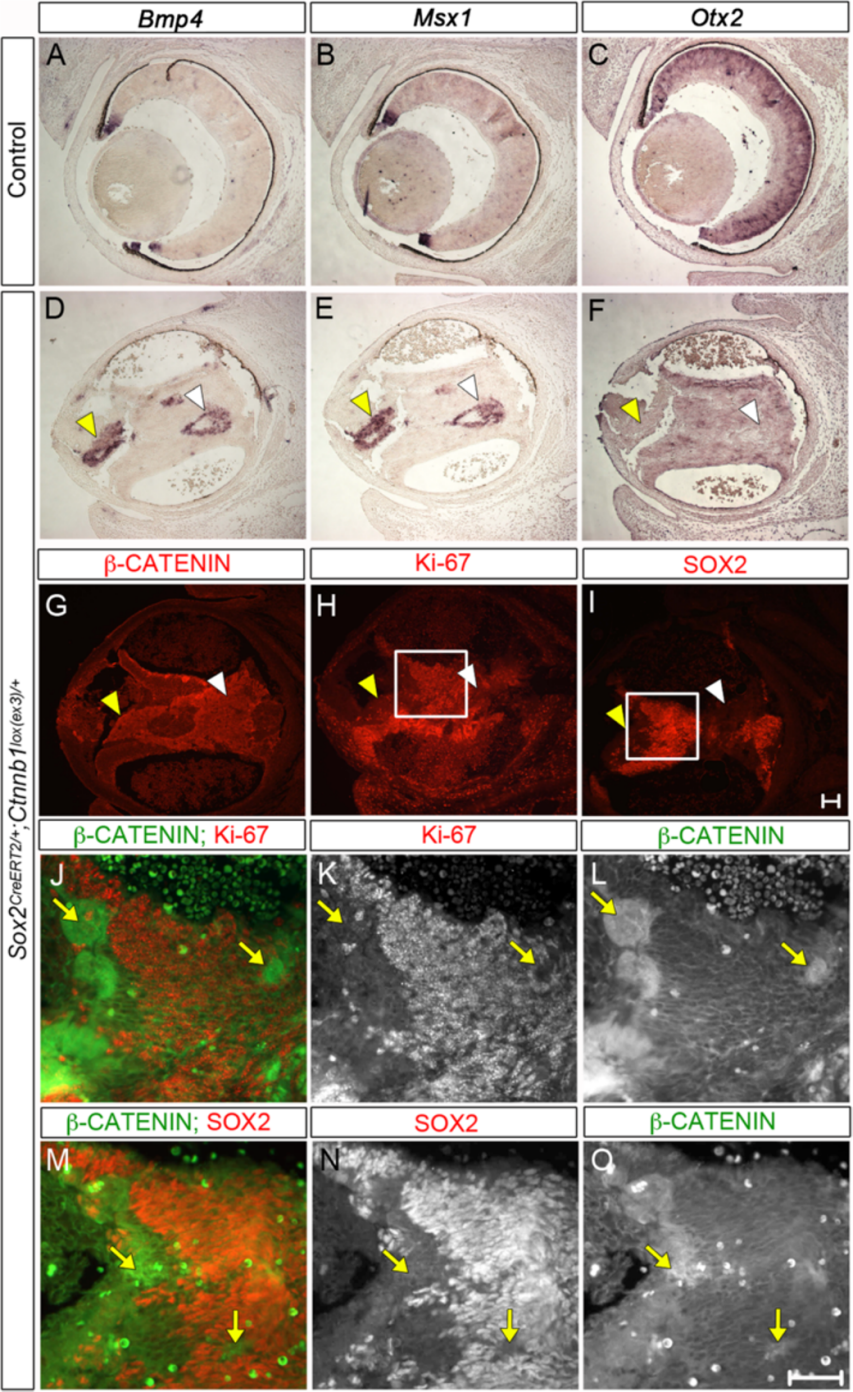
**Expression of an activated mutant form of** ***Ctnnb1*** **in the neural retina (NR) induces ciliary epithelium (CE)-** **like cell fate transformation.** Transverse sections of mutant eyes induced with tamoxifen to express *Ctnnb1* ^*lox*(*ex3*)^ in *Sox2*-expressing cells at E11.5 were analyzed at E15.5. **(A-I)** *In situ* hybridization for *Bmp4* **(A, D)** and *Msx1* **(B, E)** shows specific expression of these CE genes in ectopic regions, which accumulate β-Catenin in serial sections **(G)**. Loss of NR fate is evident through *in situ* hybridization for *Otx2* **(F)** normally marking the NR **(C)**. Yellow and white arrowheads indicate two regions of β-Catenin accumulation across the different markers in serial sections **(D-I)**. The cell fate transformation is accompanied by loss of proliferative capacity as seen by anti-Ki67 immunofluorescence **(H)** and loss of SOX2 **(I)**. **(J-O)** Double immunofluorescence for β-Catenin and Ki67 **(J-L)** and β-Catenin together with SOX2 **(M-O)** reveal that areas of β-Catenin accumulation in induced *Sox2* ^*CreERT2*/+^;*Ctnnb1* ^*lox*(*ex3*)/+^ retinas are accompanied by the loss of proliferative capacity and the specific reduction of SOX2 indicating loss of NR fate. Two representative regions of β-Catenin accumulation are indicated across the three markers with yellow arrows (panels **J-** **O**). Scale bars: 200 μm.

To confirm the notion that up-regulation of the WNT pathway is sufficient to cause the NR-to-CE fate transformation observed in *Sox2* mutants, we used a complementary approach, expressing a stable mutant form of *Ctnnb1* specifically in NR-committed *Sox2*-expressing cells. *Sox2*^*CreERT2*/+^ males [47] were crossed with *Ctnnb1*^*lox*(*ex3*)/*lox*(*ex3*)^ females, which were injected with tamoxifen at E11.5 to activate Cre expression. Analysis of *Sox2*^*CreERT2*/+^;*Ctnnb1*^*lox*(*ex3*)/+^ mutant eyes at E15.5 revealed abnormal NR morphology showing signs of detachment (Figure 4). We observed ectopic areas of activation of the CM genes *Bmp4* and *Msx1* (Figure 4D, E), which corresponded to regions accumulating β-Catenin, as seen on serial sections (arrowheads, Figure 4G), and lacking the bipolar cell and photoreceptor marker *Otx2* (Figure 4F) [48]. These areas also showed gross reduction in SOX2 protein (Figure 4I) and Ki67 (Figure 4H), further suggestive of loss of NR fate and consistent with a reduction in proliferative capacity in the CE. Of note, not all regions accumulating β-Catenin activated expression of CE genes; however, upon close examination, it was evident that SOX2 and Ki67 were excluded from β-Catenin-accumulating cells (arrows, Figure 4J-O). Taken together, these data suggest that WNT pathway activation can override establishment of NR fate even in cells initially expressing *Sox2*. Despite WNT activation being a consequence of SOX2 loss, stable expression of *Ctnnb1* resulted in a subsequent reduction in SOX2 expression.

### Attenuation of WNT signaling does not alter RPC proliferation

**Figure 5:**
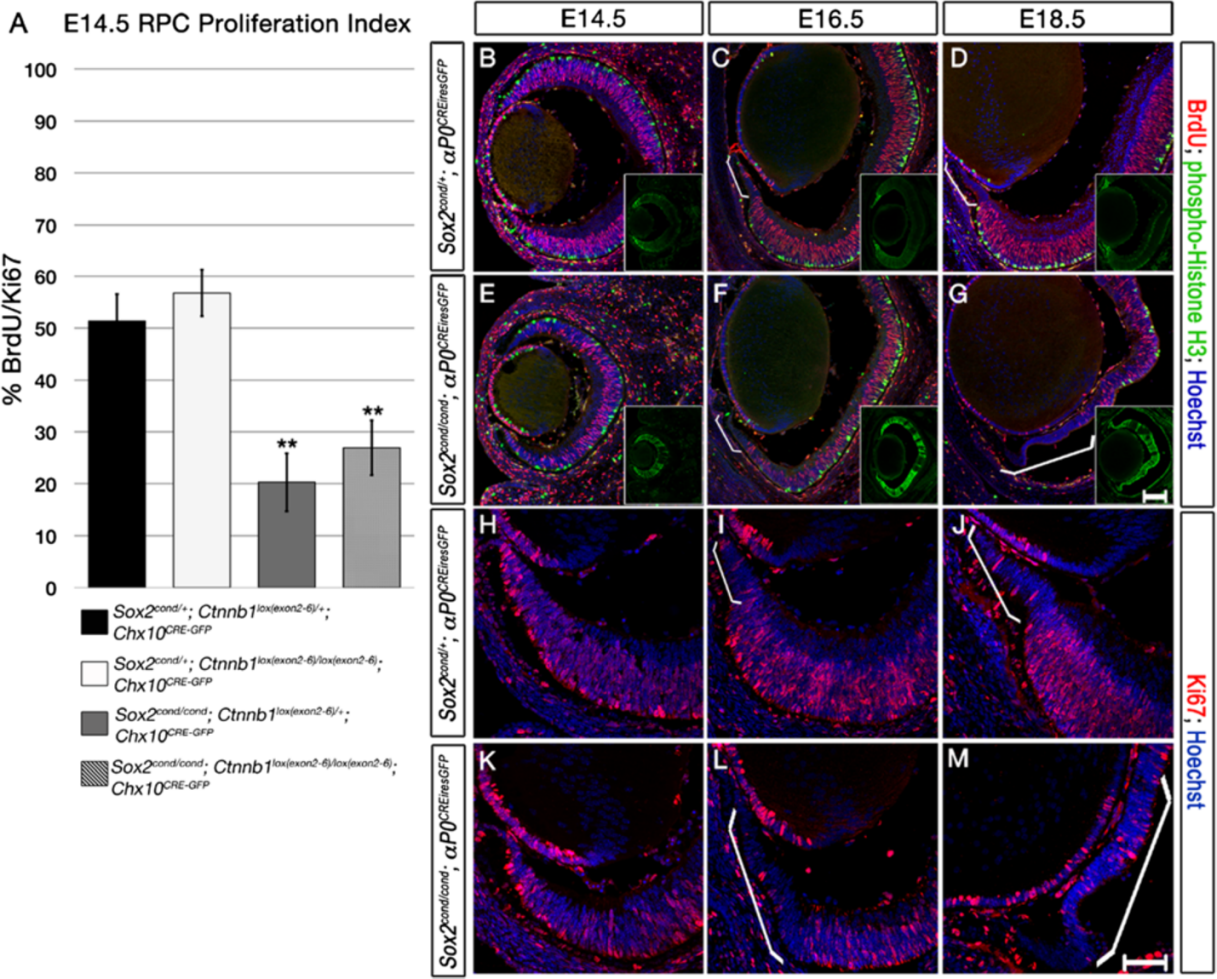
***Sox2-*** **ablated optic cup progenitor cells (OCPCs) exhibit** ***Ctnnb1-*** **independent proliferation defects.** BrdU incorporation of cycling cells was quantified in single-mutants, double-mutants and wild-type controls after a single pulse at E14.5, and OCPC proliferation in wild-type controls and *Sox2* single-mutants was assessed at 3 developmental time-points. **(A)** No significant difference in the BrdU/Ki67 index between wild-types and *Ctnnb1* ^*lox*(*ex2*–*6*)/*lox*(*ex2*–*6*)^ single-mutants at E14.5 is detected, but *Sox2* single-mutants show significantly reduced BrdU incorporation. There is no significant difference in BrdU incorporation between *Sox2* single-mutants and *Sox2*/*Ctnnb1* double-mutants. **(B-G)** Incorporation of BrdU and concomitant staining for phospho-Histone-H3 between E14.5 and E18.5. Normal ciliary epithelium (CE) development from E14.5-E18.5 is characterized by a gradual reduction in BrdU incorporation over time (**B-D**, white brackets in **C** and **D**). In *Sox2* single-mutants, the peripheral BrdU-negative region expands over time (**E-G**, white brackets in **F** and **G**). **(H-J)** Ki67 is maintained in the peripheral optic cup (OC) of controls at E14.5 **(G)** but is increasingly reduced at E16.5 **(H)** and E18.5 **(I)**. **(K-M)** Ki67 expression in the peripheral OC of *Sox2*-mutants is similar to that of controls at E14.5 **(J)** but is increasingly reduced in the periphery at E16.5 **(K)** and E18.5 **(L)**. Insets in **(A** **-F)** show GFP staining to indicate where Cre was expressed. Scale bars: 50 μm.

Down-regulation of WNT signaling was not sufficient to improve *Sox2*-mutant microphthalmia (Figure 3). Moreover, activation of WNT signaling led to a simultaneous reduction in both SOX2 and Ki67 expression (Figure 4). We therefore hypothesized that SOX2 regulation of RPC proliferation occurs independently of WNT activation. This contrasts the finding that WNT signaling promotes proliferation of embryonic stem cell-derived RPCs upstream of *Sox2* [49]. Alternatively, widespread WNT activation *in vivo* at an early developmental time point (E11.5) may have a different effect than that of WNT activation *in vitro* in differentiating neurons. To analyze the roles of SOX2 and WNT signaling in RPC proliferation *in vivo*, we quantified BrdU incorporation in single-mutant, double-mutant and control eyes over two hours following a single injection at E14.5 (Figure 5).

By two-factor analysis of variance (ANOVA), *Sox2* deletion showed a significant effect on percent BrdU incorporation (51.3% and 56.8% for wild-type controls and *Ctnnb1* single-mutants versus 20.3% and 26.9% for *Sox2* single-mutants and double-mutants, respectively), F(1,15) = 114.95, *P* < 0.001 (Figure 5A). Genetic deletion of *Ctnnb1* alone had no significant effect, F(1,15) = 0.52, *P* > 0.05. *Sox2*/*Ctnnb1* double-mutants did not show a significant difference in BrdU incorporation compared with *Sox2* single-mutants, suggesting that β-Catenin-mediated WNT signaling may not act in synergy with SOX2 in the regulation of RPC proliferation, F(1,15) = 4.41,*P* > 0.05. Together, these data demonstrate that removing *Ctnnb1* has little effect on RPC proliferation in both wild-type and *Sox2*-mutant contexts.

### Ablation of SOX2 leads to increased cell cycle time

Cell cycle exit and the onset of neuronal differentiation are tightly coupled. Given the proliferation and neurogenesis defects of the *Sox2*-mutant OC, we further characterized cell cycle dynamics of control and mutant eyes by examining the patterns of BrdU incorporation, phospho-Histone-H3 and Ki67 expression in wild-type and *Sox2*-mutant eyes over time (E14.5-E18.5; Figure 5B-M). BrdU incorporation was observed in RPCs from the center to the periphery of control OCs; however, at E14.5, fewer cells in the periphery of *Sox2*-mutant OCs incorporated BrdU compared with controls (Figure 5E versus B). This peripheral region of decreased BrdU incorporation expanded centrally at E16.5 and E18.5 (Figure 5F, G versus C, D). By contrast, at E14.5, there was no difference in the pattern of Ki67 expression between control and mutant OCs (Figure 5K versus H). However, at E16.5, the Ki67-depleted peripheral region was expanded in mutants compared with controls (Figure 5L versus I), and this expansion further increased by E18.5 (Figure 5M versus J). By P0, the Ki67-negative region was extended far into the center of the *Sox2*-mutant OC (Additional file 4A-C versus D-F, area between arrowheads). Notably, some central *Sox2*-ablated cells still expressed Ki67 at P0, as did wild-type CE cells just adjacent to the NR of control eyes (Additional file 4B, C versus E, F arrows).

**Figure 6:**
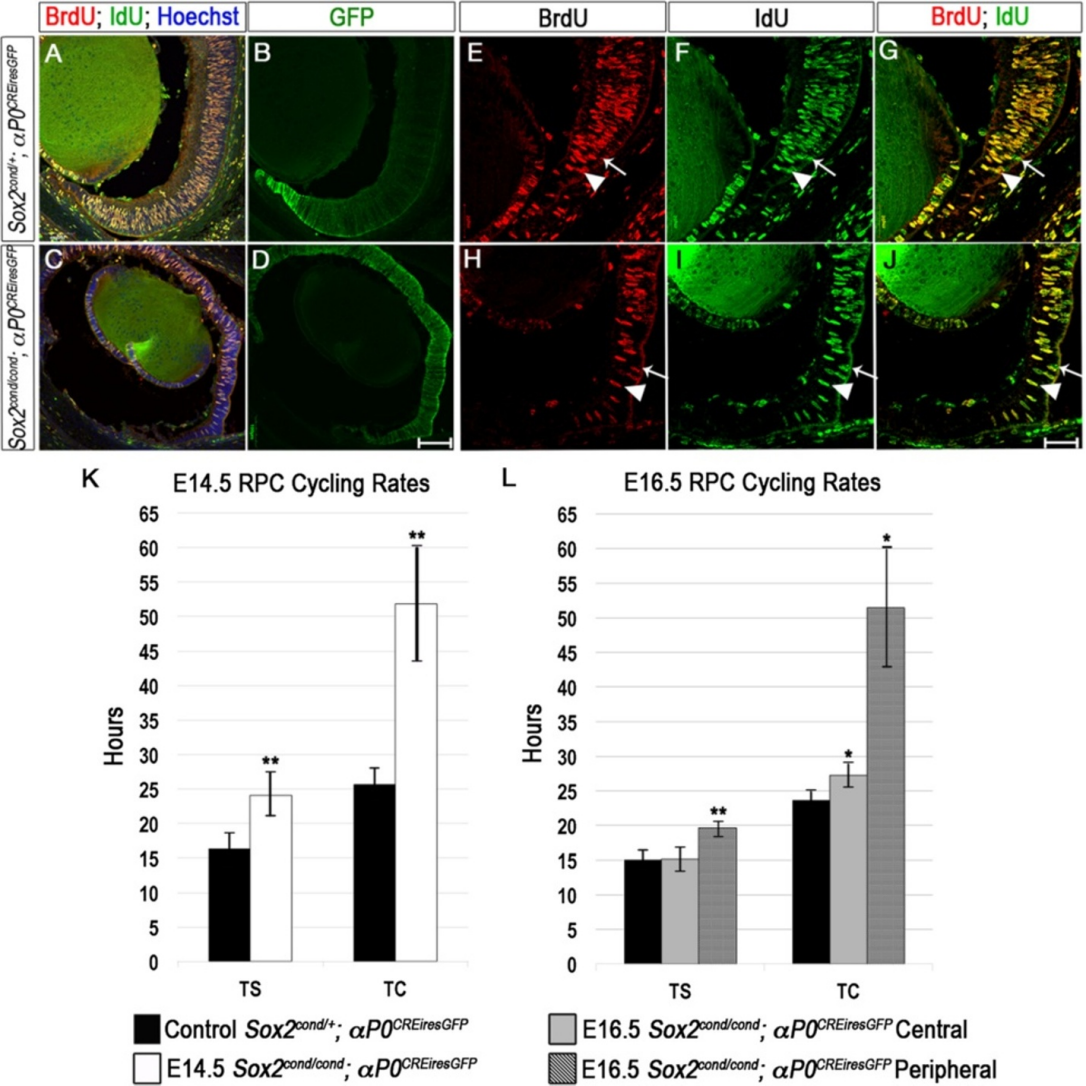
***Sox2-*** **mutant retinal progenitor cells (RPCs) take longer to complete the cell cycle.** TS and TC were quantified in control and *Sox2* single-mutant RPCs at E14.5 and E16.5. **(A-J)** Co-labeling with BrdU and IdU indicates which cells have finished S-phase during the protocol (green only, arrows) and which cells remain in S-phase (yellow, arrowheads) in wild-type **(A-G)** and mutant **(C-J)** optic cups (OCs). **(K, L)** Both TS and TC are significantly longer in mutant RPCs compared with controls at E14.5 **(K)**. AT E16.5, TS and TC vary between central and peripheral mutant RPCs **(L)**. TS is significantly longer in peripheral RPCs of mutants compared with controls. TC is significantly longer in both central and peripheral RPCs of mutants compared with controls. **(E-J)** are high power magnifications of **(A-D)**. Scale bars: **D** (for **A-D**): 100 μm; J (for E-J): 50 μm.

These observations raise two non-mutually exclusive possibilities: 1) *Sox2*-mutant cells prematurely exit the cell cycle and/or 2) increase total cell cycle length in a peripheral-to-central gradient. To more precisely characterize the cell cycle dynamics of *Sox2*-ablated cells, we quantified the lengths of S-phase (TS) and cell cycle (TC) at E14.5 and E16.5 by pulse-chase labeling with thymidine analogs (Figure 6A-J) [50]. At E14.5, there was a marked difference in the TS of central mutant RPCs compared with controls (24.08 versus 16.30 hours, respectively, *P* = 0.0002; Figure 6K). TC was also increased in mutants compared with controls (51.84 versus 25.51 hours, respectively, *P* = 0.00002). At E16.5, there was an additional difference in cell cycle dynamics within *Sox2*-mutant RPCs but not within controls. More peripheral RPCs of mutants appeared to cycle more slowly than those toward the center of the OC. We therefore divided the E16.5 mutant OC into 'central' and 'peripheral' bins to quantify the difference in cell cycle rate between the two regions (Figure 6L). Notably, for central RPCs, the difference in TS between control and GFP+ mutant cells was not significant (15.08 versus 15.16 hours, respectively, *P* = 0.9), but the difference in TC was significant (23.68 versus 27.22 hours, respectively, *P* = 0.007). Moreover, both TS and TC were significantly longer in peripheral RPCs of mutants compared with controls (TS: 19.65 versus 15.08 hours, respectively, *P* = 0.0002 and TC: 51.44 versus 23.68 hours, respectively, *P* = 0.002).

Taken together, these findings suggest that *Sox2*-mutant RPCs increase cell cycle time and exit the cell cycle in a graded manner from the periphery to the center. At E16.5, central mutant RPCs appear to be unaffected by, or perhaps compensate for, loss of SOX2 and progress through S-phase at the proper rate.

### D-type Cyclins are increased in *Sox2*-mutant cells

**Figure 7:**
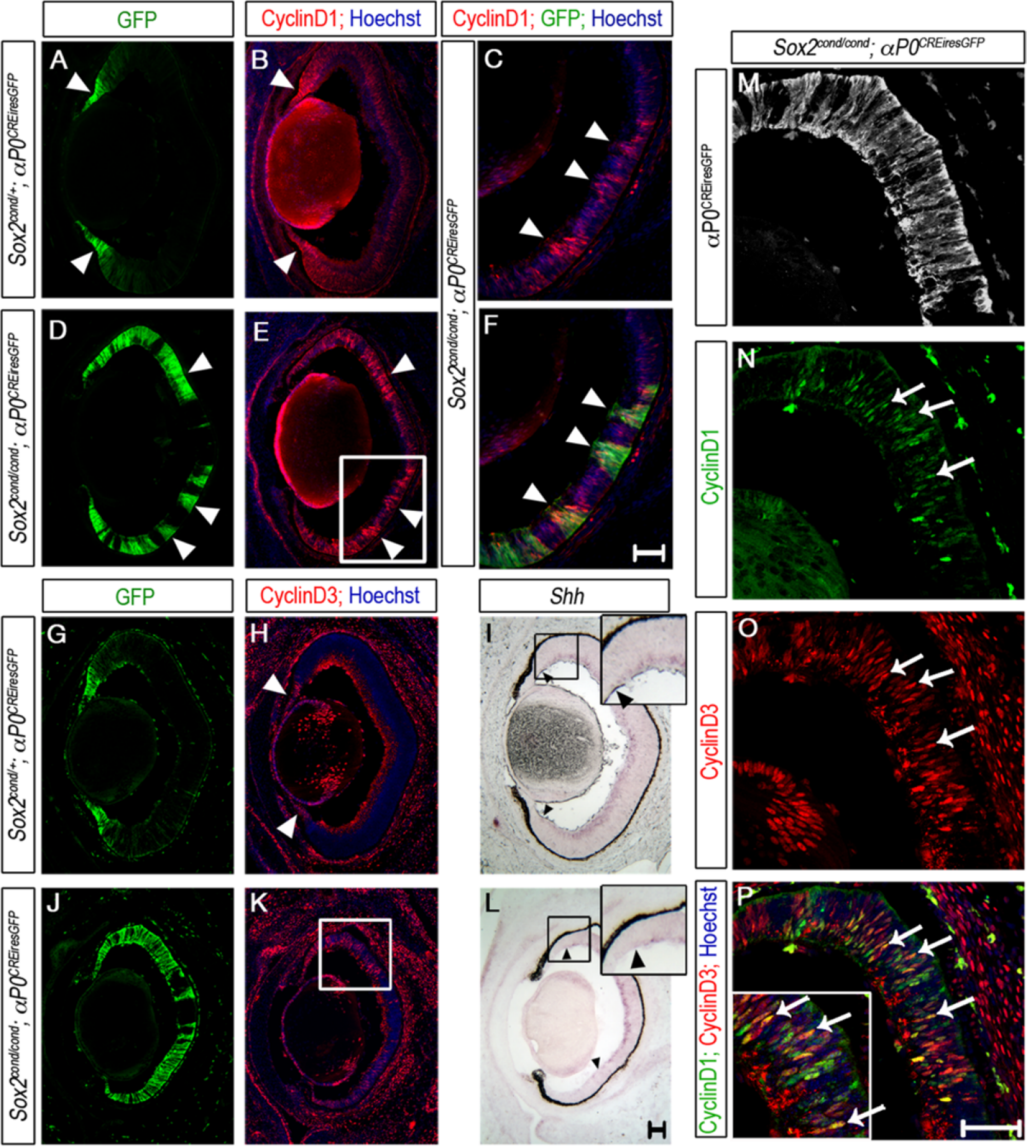
**D-** **type Cyclins are aberrantly expressed in** ***Sox2-*** **ablated optic cup progenitor cells (OCPCs) at E16.5.** Expression of D-Cyclins and *Shh* was assayed on transverse sections through E16.5 eyes. **(A-F)** CyclinD1 protein is increased in GFP-positive/*Sox2*-ablated cells (**C-F** arrowheads) compared with controls **(A, B)**. High power magnification of the boxed area in **(E)** shows cell-autonomous up-regulation of CyclinD1 **(C, F)**. CyclinD1 protein is also elevated in the peripheral NR of controls (arrowheads in **A**, **B**). **(G-** **H, J-** **K)** CyclinD3 is restricted to the peripheral OC/prospective ciliary epithelium (CE) of controls (**G, H**, arrowhead) and is ectopically expressed in central mutant OCPCs (box in **K**), supporting NR-to-CE cell fate conversion. Note that speckled staining in the inner region of the optic cup visible in **(H, K, O, P)** is non-specific background. **(I, L)** *Shh* mRNA expression is decreased in central OCPCs of mutants **(L)** compared with controls **(I)**. **(M-P)** High power magnification of the boxed area in **(K)** shows that CyclinD1 and CyclinD3 are aberrantly co-expressed in some *Sox2*-mutant central OCPCs (arrows in **P**). Black arrowheads in **(I)** and **(L)** indicate the extent of *Shh* expression. The boxed regions in **(I)** and **(L)** are magnified twice in the inserts in each panel. Scale bars: 50 μm.

Despite losing neural competence, *Sox2*-mutant RPCs continue to cycle well after deletion of *Sox2*. We next asked how RPC proliferation is supported in the absence of SOX2. D-type Cyclins are expressed in RPCs and promote progression through the cell cycle [51–53]. We examined expression of CyclinD1 and CyclinD3 protein by immunofluorescence in control and mutant OCs at E16.5 and P0. In control embryos, CyclinD1 was localized to the prospective NR, with higher levels at the boundary of the NR and CE and extending into the presumptive CE (Figure 7A, B arrowheads) as observed in zebrafish [11]. Notably, CyclinD1 protein was increased in some *Sox2*-negative (GFP-positive) RPCs (Figure 7D, E arrowheads). This increase appeared cell-autonomous in regions where Cre expression was mosaic (Figure 7C, F arrowheads). CyclinD3 protein was confined to the prospective CE of controls but expanded into the central OC of mutants (Figure 7G, H versus J, K). Co-labeling with CyclinD1 and CyclinD3 indicated that these two proteins, which are normally mutually exclusive, were aberrantly co-expressed in some *Sox2*-depleted cells (Figure 7M-P; arrows) [53].

Sonic Hedgehog (SHH) regulates RPC proliferation in part through CyclinD1 [54–57]. To address whether an increase in SHH at E16.5 could explain the increase in CyclinD1 protein, we analyzed *Shh* mRNA levels by microarray and ISH. *Shh* expression was decreased 4.5-fold in mutant retinas compared with controls (*P* = 0.0014), and the decrease was confirmed by ISH (Figure 7I versus L). The primary source of SHH in the developing retina is thought to be retinal ganglion cells (RGCs), as we have also shown by ISH at E13.5 [58] (Figure 2). Given that *Sox2*-ablated OCs generate far fewer RGCs and thus have decreased *Shh* expression, elevated CyclinD1 protein in *Sox2*-ablated cells is most likely driven by an additional mitogenic pathway not involving SHH.

### CyclinD1 expression in the retina is independent of WNT and PAX6 signaling

We next queried if aberrant CyclinD1 expression is due to WNT activation given that *Ccnd1* is a major transcriptional target of β-Catenin [59]. To address whether ectopic WNT activity drove the increase in D-Cyclins observed in *Sox2*-mutant OCs, we examined expression of D-Cyclins in single-mutant, double-mutant and wild-type control eyes at P0 (Additional file 5). CyclinD1 was expressed in GFP-positive central OCPCs in wild-type and *Ctnnb1* single-mutant eyes (Additional file 5A-B, E-F, I- J). As expected, CyclinD1 protein was increased in central RPCs of P0 *Sox2* single-mutants (Additional file 5C, G, K; arrows). Moreover, CyclinD1 remained increased even after deletion of *Ctnnb1* (Additional file 5D, H, L; arrows), suggesting that retinal CyclinD1 levels are completely independent of *Ctnnb1*. CyclinD3 protein was restricted to the CE of both wild-type and *Ctnnb1* single-mutant eyes at P0, and it was ectopically expressed in the central OC of *Sox2* single-mutants and *Sox2*/*Ctnnb1* double-mutants (Additional file 5M-T). Collectively, these results suggest that SOX2 antagonizes D-Cyclins in the OC independently of WNT/β-Catenin signaling.

The paired-box transcription factor PAX6 is crucial for maintaining RPC proliferation and multipotency [60, 61]. Despite increased cell cycle exit and decreased *Ccnd1* transcription in *Pax6*-depleted cells, CyclinD1 protein remained elevated, suggesting that PAX6 regulates CyclinD1 expression post-transcriptionally or post-translationally. This study thus revealed a complex role for PAX6 in the regulation of RPC proliferation and CyclinD1 expression [62]. To test the alternative hypothesis, that increased PAX6 protein in the *Sox2*-ablated OC was responsible for the elevated CyclinD1, we genetically decreased *Pax6* in *Sox2*-ablated RPCs using *Pax6*^*Sey*/+^ (small eye) mice as previously described [5]. The *Pax6*^*Sey*/+^ allele contains an early nonsense mutation in the *Pax6* locus, resulting in a truncated protein widely used as a *Pax6*-null [63, 64]. We compared CyclinD1 expression in S*ox2*^*cond*/*cond*^;*Chx10*^*CreGFP*^ single-mutant OCs with *Sox2*^*cond*/*cond*^;*Pax6*^*Sey*/+^;*Chx10*^*CreGFP*^ double-mutant OCs and *Pax6*^*Sey*/+^ single-mutant and wild-type controls at E14.5 (Additional file 6). As expected, CyclinD1 protein was elevated in central *Sox2* single-mutant cells when compared with wild-type and *Pax6* single-mutant cells (Additional file 6E, F versus A-D). Moreover, CyclinD1 protein remained up-regulated in *Pax6*/*Sox2* double-mutant OCPCs in the central OC. Elevated CyclinD1 protein was therefore independent of PAX6 expression in the *Sox2*-mutant OC (Additional file 6G, H).

Collectively, our data indicate that loss of *Sox2* in the developing OC resulted in a prolonged cell cycle and eventual cell cycle exit. During mid-retinogenesis (E16.5), centrally located *Sox2*-mutant OCPCs appeared to cycle at the normal rate and exhibited WNT- and PAX6-independent increased CyclinD1. By contrast, the NR-to-CE primordium cell fate conversion observed upon ablation of *Sox2* was regulated by WNT/β-Catenin signaling. The prevention of ectopic CE/CM gene expression by removal of *Ctnnb1* from *Sox2*-mutant cells, but not of increased D-Cyclin proteins, supports the conclusion that β-Catenin is a major regulator of CE fate but not of OCPC proliferation.

## Discussion

### SOX2 regulates the neurogenic/non-neurogenic boundary of the retina through modulation of WNT signaling

SOX proteins have been shown to physically interact with β-Catenin to modulate the expression of WNT target genes [24, 38–40]. The SOX2 HMG domain is not required to inhibit β-Catenin-induced gene expression in osteoblasts, suggesting that SOX2 antagonizes WNT signaling through its C-terminus, most likely via β-Catenin sequestration [24]. Alternatively, SOX2, distantly related to the TCF/LEF family of transcription factors, which also bind DNA via an HMG domain, could compete with TCF/LEF for direct DNA binding [65].

Our data support a model in which SOX2 and WNT signaling intersect at the level of target gene expression. It is still unclear, however, how SOX2 antagonizes CE fate. Using genetic epistasis analysis, we demonstrated that SOX2 and canonical WNT signaling coordinate opposing OC fates (modeled in Additional file 7). Both *Sox2* LOF and *Ctnnb1* GOF resulted in expansion of CE at the expense of NR, which is a direct consequence of elevated WNT signaling, as we find an up-regulation of WNT response in *Sox2* LOF mutants. In line with this, *Ctnnb1* LOF resulted in partial restoration of NR fates in *Sox2* LOF eyes, where CE-specific genes failed to become up-regulated, demonstrating that regulation of CE identity by SOX2 is mediated by WNT signaling. However, the neurogenesis defect observed in *Sox2* LOF mutants was not restored by limiting activation of the WNT pathway, suggesting a WNT-independent role of SOX2 in the regulation of neurogenic fates. Our data reveal that precise coordination of SOX2 and WNT signaling is necessary to ensure proper development of the neurogenic retina and circumjacent CB.

### SOX2 regulates the cell cycle independently of the WNT pathway

Genetic ablation of *Sox2* in the OC resulted in prolonged RPC cell cycle times. This increase is in line with a fate transition of the NR to CE, where cells have a longer cell cycle [4, 7]. In the *Xenopus* OC, SOX2 was found to be necessary for both proliferation and neural competence, agreeing with the findings of our study [17]. SOX2 was shown to perform this function downstream of WNT while also feeding back on the WNT pathway to inhibit it, thus painting a more complex picture of WNT-SOX2 interactions in OC development [17, 25].

Our findings indicate that even a modest increase in WNT activity upon SOX2 inactivation is associated with reduced proliferation. Reduced proliferation following WNT activation has been seen previously [8, 11, 66] and aberrant WNT activity was associated with restricted regenerative capacity in the planarian *Procotyla fluviatilis* [67]. Despite WNT activation in *Sox2*-mutant OCs, the cell cycle defect of *Sox2*-mutant cells was not rescued through deletion of *Ctnnb1* and successful down-regulation of the WNT pathway. Conversely, expression of activated *Ctnnb1* in *Sox2*-expressing RPCs promoted CE fate and reduced Ki67 only after a dramatic loss of SOX2 in cells accumulating β-Catenin, which was not investigated in previous reports [8, 11, 25]. Our experimental evidence thereby supports a model in which SOX2 regulates RPC proliferation independently of WNT signaling in the mouse.

### The *Sox2*-mutant phenotype suggests complex regulation of CyclinD1 by SOX2

It was somewhat surprising that RPCs continued to proliferate well after loss of SOX2. One possible explanation for this sustained proliferation is that stabilized D-Cyclins permitted OCPCs to progress through the cell cycle. These data raise an additional contradiction: increased Cyclins would not be expected to correlate with the observed increase in cell cycle time and decreased BrdU incorporation. Nonetheless, in support of our finding, at least one other instance of increased CyclinD1 protein and decreased proliferation has been observed in the OC: *Pax6*-ablated OCPCs showed elevated CyclinD1 protein and increased cell cycle exit. In this context, CyclinD1 was not sufficient to maintain the proliferation of *Pax6*-ablated cells, since the CyclinD1-positive cells did not incorporate BrdU [62]. Thus, CyclinD1 expression may not indicate sustained proliferation. Moreover, *Sox2*-ablated cells undergo a cell fate transformation from a more proliferative to a less proliferative cell type (NR-to-CE). The presence of high CyclinD1 protein at the CM at E16.5 (Figure 7b, arrowhead) raises the possibility that cells normally undergoing the NR/CE cell fate decision have elevated CyclinD1 levels [68].

The decrease in *Ccnd1* mRNA suggests that CyclinD1 transcription is positively regulated by SOX2, as SOX2 has been shown to be capable of transactivating the *Ccnd1* promoter *in vitro* [39]. The unexpected negative regulation of CyclinD1 protein may take place through an alternative cascade influenced by SOX2. In human embryonic stem cells (ESCs), SOX2 and OCT4 promote the expression of miR-302, a cluster of 8 microRNAs expressed in pluripotent cells, and miR-302a post-transcriptionally represses *Ccnd1* [69]. Moreover, OC-specific ablation of *Dicer1*, a major mediator of microRNA biosynthesis, resulted in increased CyclinD1 protein in mouse OCPCs, creating a persistent CM-like region [70]. Therefore, even with decreased *Ccnd1* transcription, CyclinD1 protein could be stabilized upon the loss of microRNA biosynthesis influenced by the lack of SOX2.

Lastly, SOX2 could antagonize CyclinD1 via direct regulation of the cell cycle inhibitor *p27*^*Kip1*^, as has been observed in inner pillar cells of the mouse auditory sensory epithelium [71]. Indeed, p27^Kip1^ protein was absent in *Sox2*-null RPCs (WEH, unpublished observations). In agreement with this notion, genetic ablation of *Sox2* specifically in the developing pituitary gland led to a severe reduction in proliferative capacity of pituitary embryonic precursors, although *p27*^*Kip1*^ expression was elevated rather than repressed [72].

### Canonical WNT signaling acts upstream of BMPs to specify CE fate

A role for BMP signaling in CE specification has been demonstrated, although the specific sources of ocular BMPs are currently unclear [34, 35, 45, 73]. Results from various studies suggest that the developing lens may instruct the peripheral OC margin to become CE [35, 73]. However, the CE is correctly specified in the absence of a lens, suggesting that the lens may be more important for maintaining CE fate [10, 74]. Our data suggest that WNT signaling within the OC acts upstream of BMP signaling such that expression of *Bmp4*/*7* and the BMP target gene *Msx1* are lost in *Ctnnb1*-mutant cells and are up-regulated with stabilization of the pathway.

### The *Sox2*-deficient OC provides a resource to identify genes important for CE development

Downstream of BMP and WNT signaling, much of CE development remains a mystery. Many of the significantly up-regulated genes in this study were associated with ion exchange and secretion (Figure 1). Up-regulation of these groups of genes is consistent with the function of the CB to maintain constant intraocular pressure (IOP) through the active transport of fluid via Na^+^-K^+^ exchange pumps and Cl^-^ channels [30]. Given that the presumptive CB epithelium significantly expands upon *Sox2* ablation, our genome-wide screen may reveal previously unidentified genes important for the function of the CB.

## Conclusions

Our study demonstrates that SOX2 regulates the neurogenic boundary of the retina in part through mediation of canonical WNT signaling, such that genetic removal of *Ctnnb1* from *Sox2*-mutant eyes partially restores neural retina cell fate. By contrast, *Sox2*, but not *Ctnnb1*, is required to maintain RPC proliferation. The increase in total cell cycle time of *Sox2*-mutant RPCs may explain how mutations in human *SOX2* contribute to microphthalmia.

## Methods

### Animals

All animal work was carried out in compliance with the University of North Carolina Institutional Animal Care and use committee (IACUC) policies and ethical approval by the Division of Laboratory Animal Medicine (DLAM) at The University of North Carolina at Chapel Hill. Generation of the *Sox2*^*Cond*/+^ mouse line was described previously [16]. *Sox2*^*cond*/+^ and *Sox2*^*cond*/+^;*αP0*^*CREiresGFP*^ mice were maintained on a C57BL/6 J background, and all others described in this study were maintained on a mixed background containing 129/Sv and C57BL/6 J. *αP0*^*CREiresGFP*^ [60], *Chx10*^*CreGFP*^ [42], *Ctnnb1*^*lox*(*ex2*–*6*)/+^ [41], *ROSA26Reporter* [43], *Pax6*^*Sey*/+^ [64], *Ctnnb1*^*lox*(*ex3*)/+^ [75] and *Sox2*^*CreERT2*/+^ [47] mice have been previously described. For CreERT2 activation, pregnant dams received a single injection of tamoxifen totaling 1.5 mg, and simultaneous injection with 0.75 mg progesterone (both Sigma-Aldrich, St Louis, MO, USA) to reduce the risk of spontaneous abortion.

### Tissue preparation, immunostaining and *in situ*hybridization

Immunostaining was performed as previously described [5]. Additional antibodies used in this study include CyclinD1 (1:400, Thermo Scientific, Waltham, MA, USA), CyclinD3 (1:100, Cell Signaling, Beverly, MA, USA), β-Catenin (1:250, Abcam, Cambridge, MA, USA) and β-galactosidase (1:10,000 Molecular Probes, Eugene, OR, USA). Antigen retrieval was used for antibodies against BrdU, CyclinD3 and phospho-Histone-H3 as previously described [76]. *In situ* hybridization was performed on 14-μm frozen sections as described [5]. The following probes were used: *Bmp4*, *Msx1*, *Ccnd1 3*’*UTR*, *Shh*, *Otx2* and *Hes5* (kind gifts from Dr A LaMantia, Dr Y Liu, Dr C Cepko, Dr E Tucker, Dr JP Martinez-Barbera and Dr E Anton, respectively). Images were captured on a Leica inverted microscope (Leica DMIRB, Leica Microsystems GmbH, Germany) equipped with a Retiga (SRV-1394, QImaging, BC, Canada) camera or on an Olympus laser scanning confocal microscope (Olympus Fluoview FV1000, Olympus America, PA, USA) and processed using Adobe Photoshop software. Unless otherwise stated, a minimum of three samples per genotype were analyzed for each assay.

### BrdU and IdU labeling

Pregnant dams (E14.5 or E16.5) were injected intraperitoneally with 120 mg IdU per kg body weight at time (T) 0. At T = 1.5 hours, dams were injected with 100 mg BrdU (Sigma B5002, Sigma-Aldrich, St Louis, MO, USA) per kg body weight. At T = 2 hours, dams were culled and embryos were fixed in cold 4% PFA. A mouse antibody against IdU and BrdU (BD) detected both thymidine analogs, and a rat antibody specific to BrdU (Serotec, Raleigh, NC, USA) detected BrdU alone [50].

### Cell counting, S-phase and cell cycle measurements

For each eye, a section through the middle of the eyecup was imaged using an Olympus Fluoview FV1000 confocal microscope (Olympus America, PA, USA). Four eyes from separate animals were collected for each genotype, for each stage. For E16.5 eyes, the prospective NR was divided into 2 bins (central and peripheral) using Olympus Fluoview 2.1c software, totaling to 2 regions per section, covering the entire prospective retina. Note, for all proliferation experiments in this study (Figures 5 and 6), we quantified data for RPCs (central OCPCs) in controls and mutants. Data for prospective CE progenitor cells (peripheral OCPCs) were too variable to support any reliable conclusions. In wild-type eyes, all proliferating cells were counted. In mutant eyes, only GFP-positive proliferating cells were counted (ranges E14.5 1,200 to 2,400 cells per section, E16.5 1,500 to 3,200 cells per section). At least 3 samples from each genotype were used in the final calculations at E16.5. For studies at E14.5, we analyzed 3 wild-type controls, 4 each from single mutant genotypes and 6 double-mutants. To determine the effect of *Sox2* and *Ctnnb1* on BrdU index, a two-factor ANOVA was performed. To determine the significance of differences in cell cycle times between controls and *Sox2* single-mutants, a Student’s *t*-test was carried out. *P*-values less than 0.05 were considered significant, and those less than 0.001 were considered highly significant. The following formula was used to calculate BrdU index:


S-phase and cell cycle times were calculated as described [50]. The ratio of 'S' cells (BrdU/IdU double-positive) to 'L' cells (IdU-only) was measured, and the following formulae were used:


### Whole-genome expression analysis

Pairs of eyes were enucleated from 6 wild-type (*Sox2*^*cond*/+^;*αP0*^*CREresGFP*^) and 6 mutant (*Sox2*^*cond*/*cond*^;*αP0*^*CREresGFP*^) embryos at E16.5 (for a total of 12 eyes per genotype). Each pair of eyes was immediately placed in RNA-Later RNA stabilization solution and stored at 4°C for no longer than 30 days. RNA was purified using an Ambion (Foster City, CA, USA) RNaqueous kit following the manufacturer’s instructions. Five hundred nanograms of RNA from each pair of eyes were amplified and biotin-labeled using an Ambion Illumina (San Diego, CA, USA) TotalPrep RNA Amplification kit. One and a half micrograms of labeled cRNA were hybridized to Illumina (San Diego, CA, USA) mouse WG-6 expression bead chips (six replicate arrays per genotype, for twelve arrays total). Microarray data were generated by Expression Analysis (Durham, NC, USA). Briefly, to detect significant changes in gene expression between controls and mutants, a permutation analysis for differential expression (PADE) was performed. PADE repeatedly randomly reassigns each sample to one of the two groups to determine if the calculated difference in levels of a transcript is non-random. PADE was also used to estimate the FDR for sets of differentially expressed transcripts. A *P*-value for each transcript was calculated using a two-sample *t*-test. Transcripts with an accumulated false discovery rate (FDR) of less than 5%, a *P*-value of less than 0.05, and a fold change of greater than ±1.6, totaling 2,194 transcripts, were chosen for further analysis. Functional gene annotation was identified using DAVID [28, 29] and potentially affected pathways were determined using Ingenuity Pathway Analysis (Qiagen, Redwood City, CA, USA).

## Electronic supplementary material

Additional file 1: **Significantly changed optic cup (OC) WNT target genes.** Thirteen known OC WNT target genes were found to be changed at least 1.47-fold (log ratio of at least ±0.55) in mutant compared with control eyes. (XLSX 640 KB)

Additional file 2: **Functional Annotation of the** ***Sox2***-**ablated optic cup (OC) reveals cell fate change.** Functional terms significantly enriched for up-regulated genes (first tab) and down-regulated genes (second tab) resulting from optic cup-specific ablation of *Sox2*. (DOCX 51 KB)

Additional file 3: **Chx10** ^**CreGFP**^ **efficiently ablates** ***Sox2*** **and** ***Ctnnb1*** **from the optic cup (OC) without causing increased cell death.** The efficiency of *Chx10* ^*CreGFP*^ and cell death were assessed in double-mutants at E14.5. (A**-**B**,** D**-**E) SOX2 and β-Catenin are absent from CreGFP-positive cells in double-mutants (C,D) compared with controls (A,B). (C,F) Cleaved Caspase 3 is similar between controls and mutants. Scale bar: 200 μm. (PNG 1 MB)

Additional file 4: ***Sox2***-**deficient OCPCs prematurely exit the cell cycle.** Cycling cells in neonatal controls and mutants were identified by Ki67 staining. (A**-**F) In controls, Ki67 is expressed in RPCs (red in A) and in some GFP-positive CE cells adjacent to the NR (B-C; arrows) but not in the most peripheral CE cells (B-C; arrowheads). In mutants, Ki67 is expressed in centrally located *Sox2*-ablated RPCs (E-F; arrows) but not in peripheral *Sox2*-ablated RPCs (E-F; arrowheads). Boxed areas in (A) and (D) are magnified in (B-C) and (E-F), respectively. Scale bars: 100 μm. (PNG 776 KB)

Additional file 5: **Deletion of** ***Ctnnb1*** **in** ***Sox2***-**ablated optic cup progenitor cells (OCPCs) does not rescue increased CyclinD1 or ectopic expression of CyclinD3.** CyclinD1 and CyclinD3 expression were analyzed in single-mutants, double-mutants and controls at P0. CyclinD1 is restricted to retinal progenitor cells (RPCs) in controls (A-C) and *Ctnnb1* single-mutants (F-H). CyclinD1 is increased in GFP-positive cells in the central OC of *Sox2* single-mutants (K-M; arrows) and in *Sox2*/*Ctnnb1* double-mutants (P-R; arrows). CyclinD3 is restricted to the CE of controls (D, E; arrows) and *Ctnnb1* single-mutants (I, J; arrows) and ectopically expressed in GFP-positive cells in the central OC of *Sox2* single-mutants (N, O; arrows) and *Sox2*/*Ctnnb1* double-mutants (S, T; arrows). Boxed areas in (A, F, K and P) are magnified in (B, C; G, H; L, M; and Q, R), respectively. Scale bars: 100 μm. (PNG 1 MB)

Additional file 6: **Reduction of** ***Pax6*** **in** ***Sox2***-**ablated optic cup progenitor cells (OCPCs) does not rescue increased CyclinD1.** CyclinD1 was assayed on transverse sections through the eyes of *Sox2* single-mutants, *Pax6* ^*Sey*/+^ single-mutants, double-mutants and wild-type controls at E14.5. (A**-**D) CyclinD1 is expressed in retinal progenitor cells (RPCs) of controls (A, B) and *Pax6* ^*Sey*/+^ single-mutants (C, D). (E**-**H) CyclinD1 is increased in central OCPCs of *Sox2* single-mutants (E, F) and *Sox2*/*Pax6* ^*Sey*/+^double-mutants (G, H). Scale bar: 200 μm. (PNG 3 MB)

Additional file 7: **Model of how SOX2 and canonical WNT signaling regulate the neurogenic boundary of the optic cup (OC).** OC-specific genetic ablation of *Sox2* or *Ctnnb1* results in complementary phenotypes. (A**-**D) The boundary between neural retina (NR) (blue) and ciliary epithelium (CE) (orange) is shifted peripherally in *Ctnnb1* single-mutants (B) compared with controls (A). Conversely, the boundary between NR and CE is shifted centrally in *Sox2* single-mutants such that WNT and BMP signaling are expanded (C) compared with controls (A). The boundary between the NR and CE remains centrally shifted in *Sox2*/*Ctnnb1* double-mutants (D). However, BMP signaling and other classical CE markers fail to be expressed in this expanded CE-like region. D-type cyclins are increased in both *Sox2* single-mutants and *Sox2*/*Ctnnb1* double-mutants. (PNG 167 KB)

## Abbreviations

ΑΝΟVΑ: analysis of variance
β-gal: β-galactosidase
BAC: bacterial artificial chromosome
BrdU: bromodeoxyuridine
CB: ciliary body
Ccnd1: CyclinD1
Ccnd3: CyclinD3
CE: ciliary epithelium
CM: ciliary margin
Ctnnb1: β-Catenin
E: embryonic day
ESCs: embryonic stem cells
FDR: false discovery rate
GFP: green fluorescent protein
GOF: gain-of-function
IdU: iododeoxyuridine
IHC: immunohistochemistry
ISH: *in situ* hybridization
IOP: intraocular pressure
LOF: loss-of-function
NR: neural retina
OC: optic cup
OCPC: optic cup progenitor cell
P: postnatal day
PADE: permutation analysis for differential expression
PCR: polymerase chain reaction
PFA: paraformaldehyde
RGC: retinal ganglion cell
RPC: retinal progenitor cell
Sey: small-eye mutation
Shh: Sonic Hedgehog
TC: cell cycle time
TGFβ: transforming growth factor beta
TS: S-phase time.
