# Supplementary material for: Establishment of the neurogenic boundary of the mouse retina requires cooperation of SOX2 and WNT signaling
Source: Neural Dev. 2014 Dec 9;9:27. doi: 10.1186/1749-8104-9-27 (PMC4295269; doi:10.1186/1749-8104-9-27)
Supplement: Supplementary file 2 — Additional file 2: Functional Annotation of the Sox2-ablated optic cup (OC) reveals cell fate change. Functional terms significantly enriched for up-regulated genes (first tab) and down-regulated genes (second tab) resulting from optic cup-specific ablation of Sox2. (DOCX 51 KB) [file 13064_2014_270_MOESM2_ESM.docx]

**Heavner et al. Additional File 2**

| **Gene Name** | **Estimated Log Ratio** | **Raw Estimated Fold Change** | **P-value** |
| --- | --- | --- | --- |
| Foxp2 | 2.61 | 6.10 | 0.0005 |
| Tcf7 | 1.95 | 3.87 | 0.0156 |
| Wfdc1 | 1.58 | 2.98 | 0.0001 |
| Tnfrsf19 | 1.38 | 2.60 | 0.0348 |
| Otx1 | 1.32 | 2.50 | 0.0001 |
| Col9a2 | 1.32 | 2.49 | 0.0000 |
| Zic1 | 1.06 | 2.08 | 0.0001 |
| Bmp7 | 0.94 | 1.91 | 0.0000 |
| Id3 | 0.59 | 1.50 | 0.0002 |
| Cdkn1a | 0.56 | 1.47 | 0.0444 |
| Sgk | 0.55 | 1.47 | 0.0027 |
| Apc2 | -1.91 | -3.75 | 0.0045 |
| Syt13 | -1.73 | -3.31 | 0.0047 |
