# Supplementary figures and images for: Establishment of the neurogenic boundary of the mouse retina requires cooperation of SOX2 and WNT signaling
Source: Neural Dev. 2014 Dec 9;9:27. doi: 10.1186/1749-8104-9-27 (PMC4295269; doi:10.1186/1749-8104-9-27)

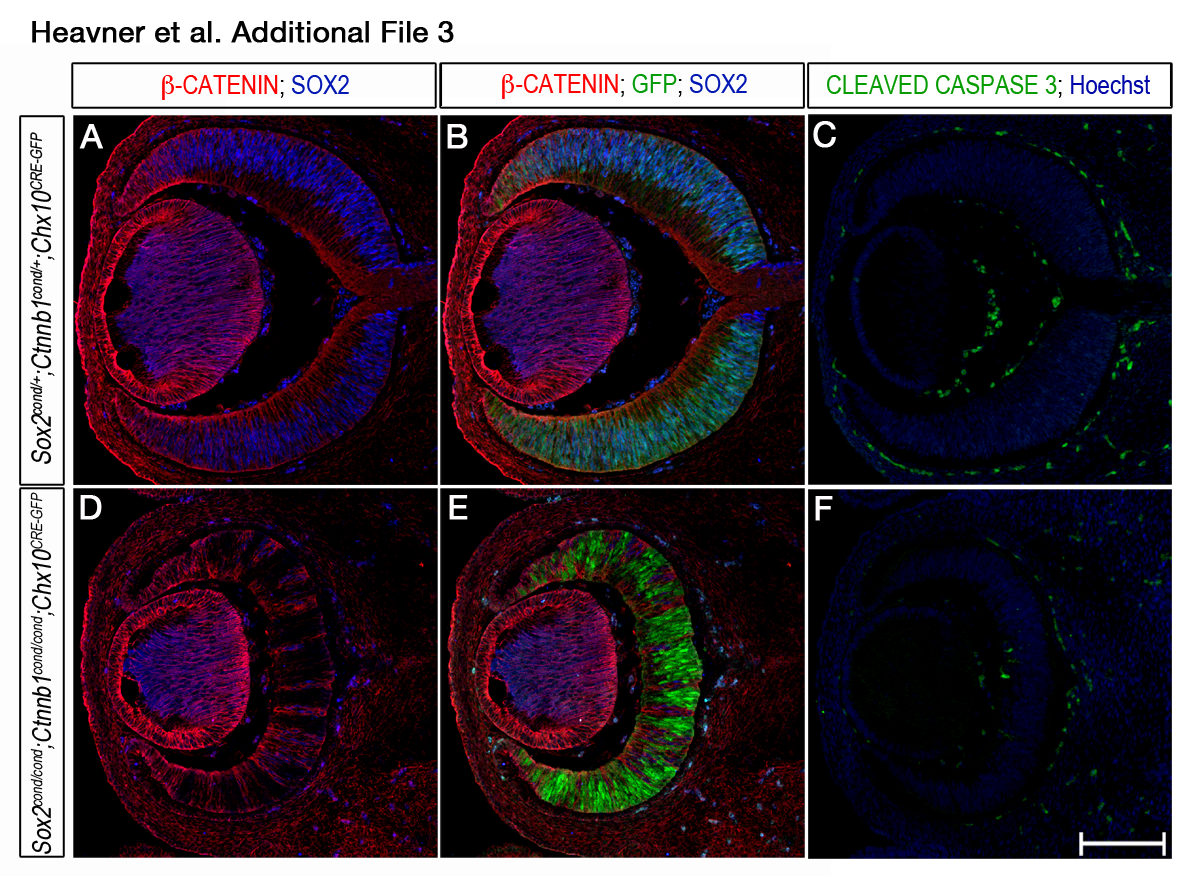

Supplement: Supplementary file 3 — Additional file 3: Chx10 CreGFP efficiently ablates Sox2 and Ctnnb1 from the optic cup (OC) without causing increased cell death. The efficiency of Chx10 CreGFP and cell death were assessed in double-mutants at E14.5. (A-B, D-E) SOX2 and β-Catenin are absent from CreGFP-positive cells in double-mutants (C,D) compared with controls (A,B). (C,F) Cleaved Caspase 3 is similar between controls and mutants. Scale bar: 200 μm. (PNG 1 MB) [file 13064_2014_270_MOESM3_ESM.png]

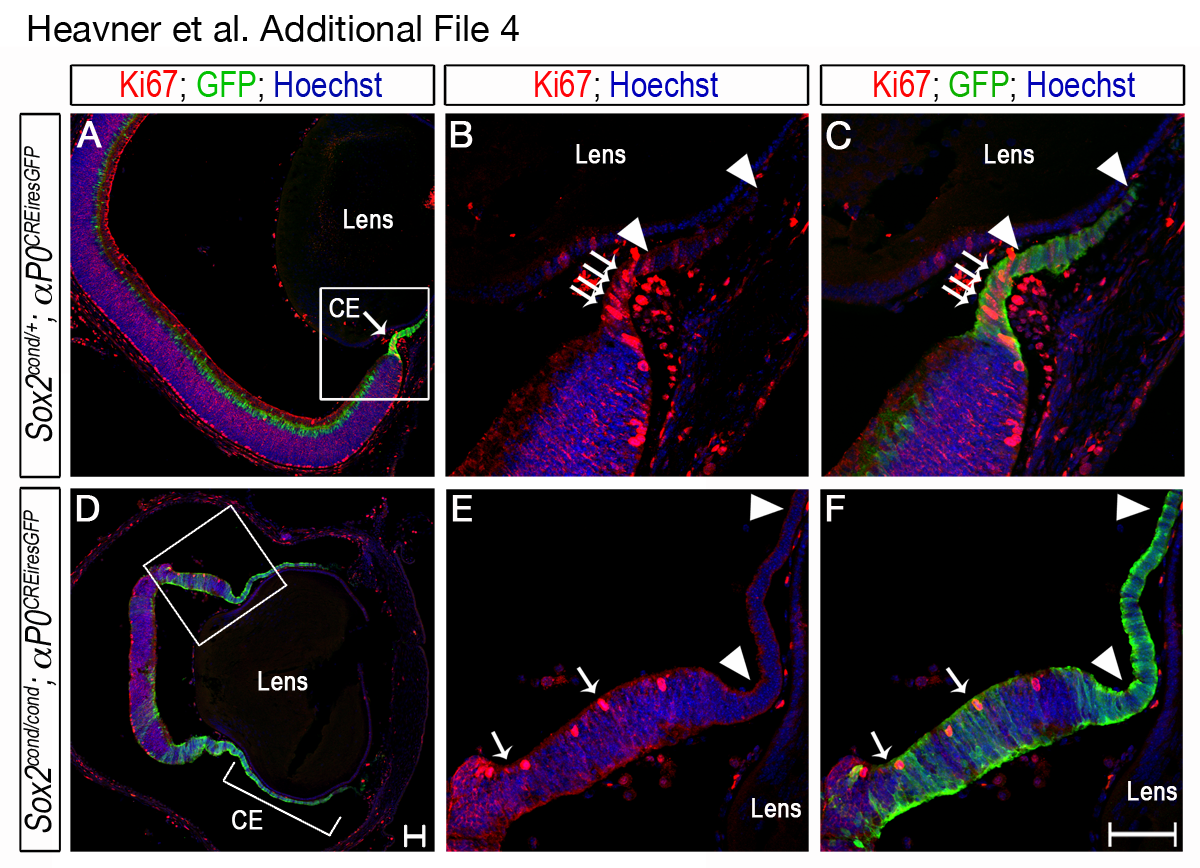

Supplement: Supplementary file 4 — Additional file 4: Sox2-deficient OCPCs prematurely exit the cell cycle. Cycling cells in neonatal controls and mutants were identified by Ki67 staining. (A-F) In controls, Ki67 is expressed in RPCs (red in A) and in some GFP-positive CE cells adjacent to the NR (B-C; arrows) but not in the most peripheral CE cells (B-C; arrowheads). In mutants, Ki67 is expressed in centrally located Sox2-ablated RPCs (E-F; arrows) but not in peripheral Sox2-ablated RPCs (E-F; arrowheads). Boxed areas in (A) and (D) are magnified in (B-C) and (E-F), respectively. Scale bars: 100 μm. (PNG 776 KB) [file 13064_2014_270_MOESM4_ESM.png]

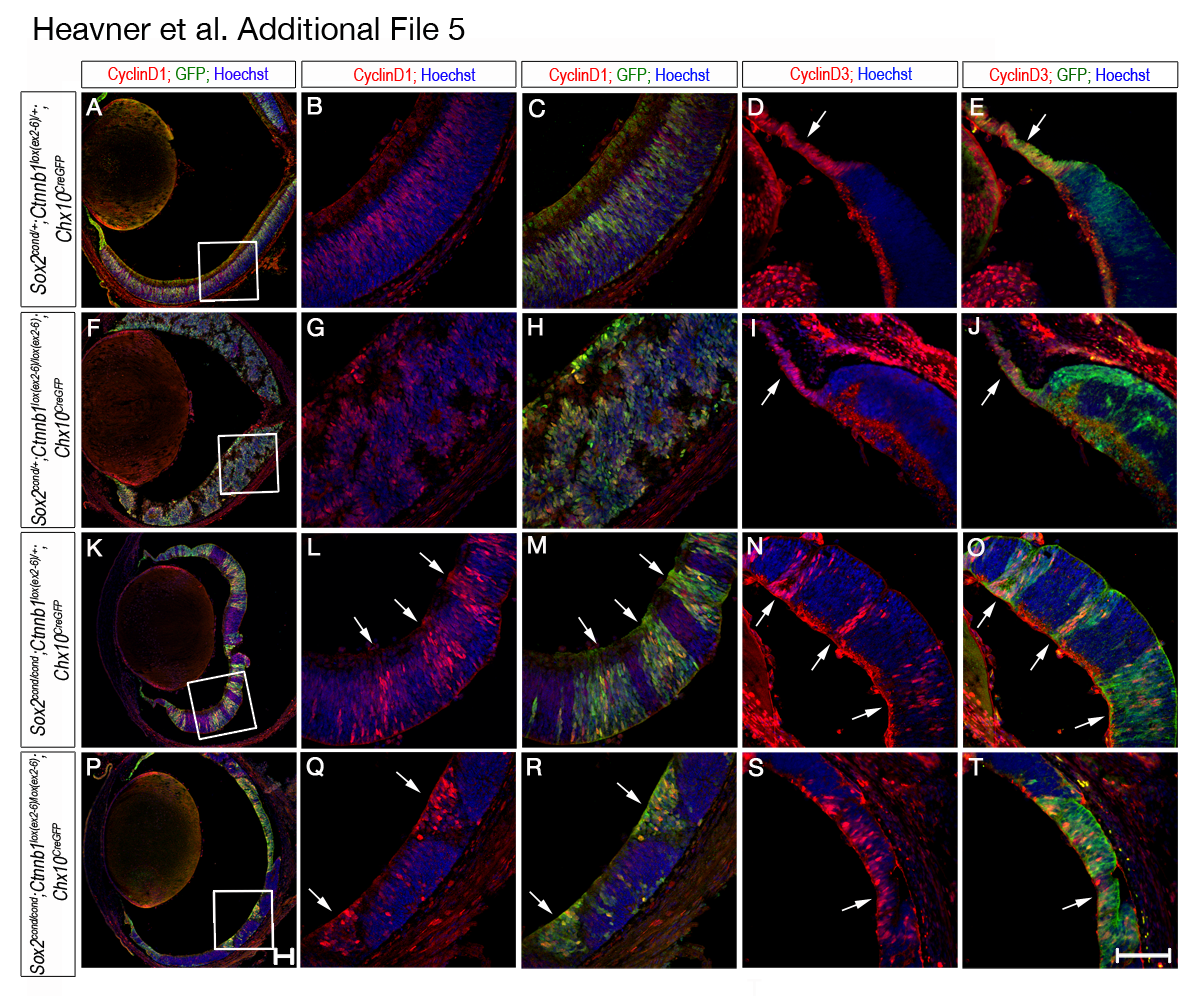

Supplement: Supplementary file 5 — Additional file 5: Deletion of Ctnnb1 in Sox2-ablated optic cup progenitor cells (OCPCs) does not rescue increased CyclinD1 or ectopic expression of CyclinD3. CyclinD1 and CyclinD3 expression were analyzed in single-mutants, double-mutants and controls at P0. CyclinD1 is restricted to retinal progenitor cells (RPCs) in controls (A-C) and Ctnnb1 single-mutants (F-H). CyclinD1 is increased in GFP-positive cells in the central OC of Sox2 single-mutants (K-M; arrows) and in Sox2/Ctnnb1 double-mutants (P-R; arrows). CyclinD3 is restricted to the CE of controls (D, E; arrows) and Ctnnb1 single-mutants (I, J; arrows) and ectopically expressed in GFP-positive cells in the central OC of Sox2 single-mutants (N, O; arrows) and Sox2/Ctnnb1 double-mutants (S, T; arrows). Boxed areas in (A, F, K and P) are magnified in (B, C; G, H; L, M; and Q, R), respectively. Scale bars: 100 μm. (PNG 1 MB) [file 13064_2014_270_MOESM5_ESM.png]

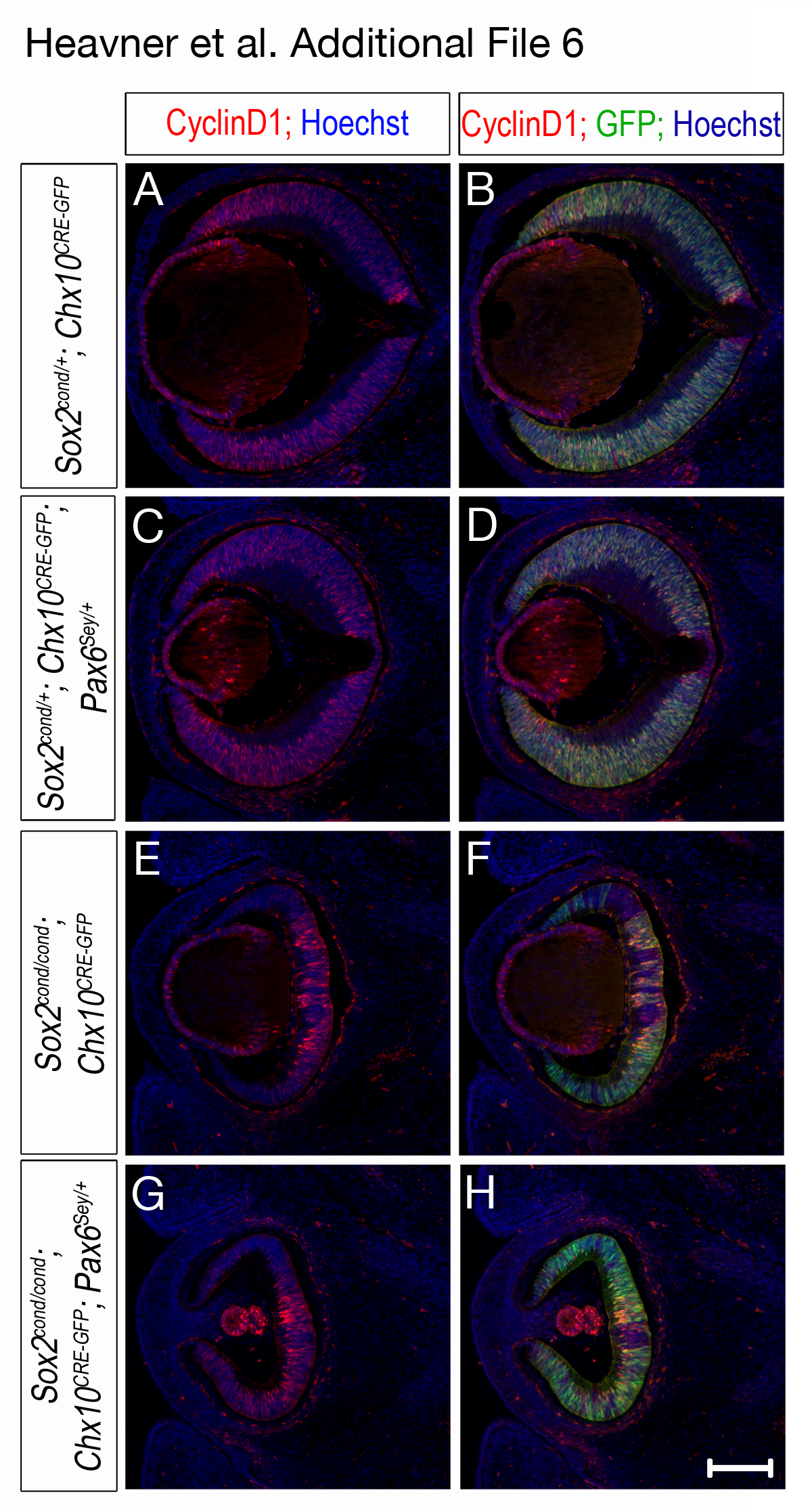

Supplement: Supplementary file 6 — Additional file 6: Reduction of Pax6 in Sox2-ablated optic cup progenitor cells (OCPCs) does not rescue increased CyclinD1. CyclinD1 was assayed on transverse sections through the eyes of Sox2 single-mutants, Pax6 Sey/+ single-mutants, double-mutants and wild-type controls at E14.5. (A-D) CyclinD1 is expressed in retinal progenitor cells (RPCs) of controls (A, B) and Pax6 Sey/+ single-mutants (C, D). (E-H) CyclinD1 is increased in central OCPCs of Sox2 single-mutants (E, F) and Sox2/Pax6 Sey/+double-mutants (G, H). Scale bar: 200 μm. (PNG 3 MB) [file 13064_2014_270_MOESM6_ESM.png]

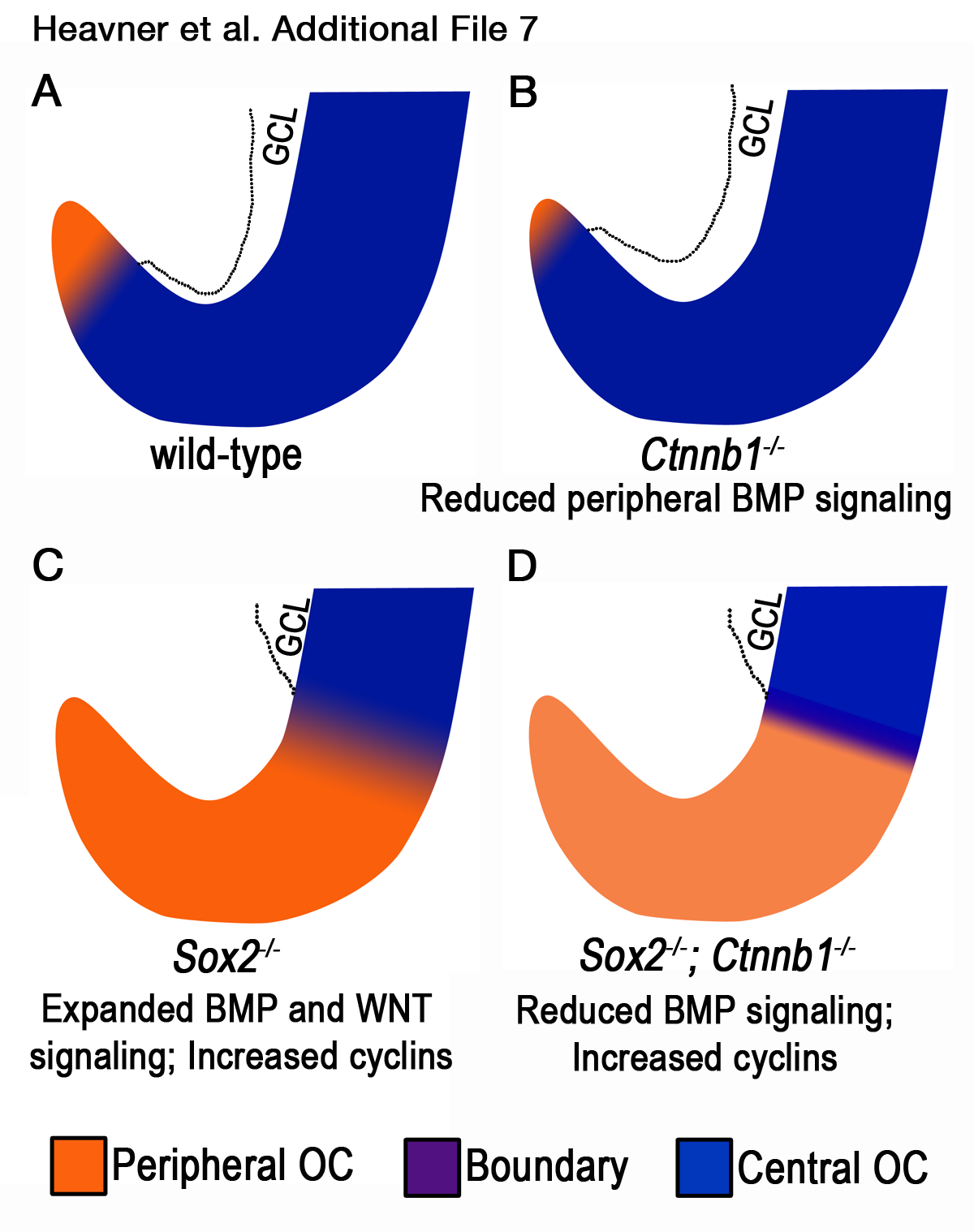

Supplement: Supplementary file 7 — Additional file 7: Model of how SOX2 and canonical WNT signaling regulate the neurogenic boundary of the optic cup (OC). OC-specific genetic ablation of Sox2 or Ctnnb1 results in complementary phenotypes. (A-D) The boundary between neural retina (NR) (blue) and ciliary epithelium (CE) (orange) is shifted peripherally in Ctnnb1 single-mutants (B) compared with controls (A). Conversely, the boundary between NR and CE is shifted centrally in Sox2 single-mutants such that WNT and BMP signaling are expanded (C) compared with controls (A). The boundary between the NR and CE remains centrally shifted in Sox2/Ctnnb1 double-mutants (D). However, BMP signaling and other classical CE markers fail to be expressed in this expanded CE-like region. D-type cyclins are increased in both Sox2 single-mutants and Sox2/Ctnnb1 double-mutants. (PNG 167 KB) [file 13064_2014_270_MOESM7_ESM.png]
